# A Macrophage-Pericyte Axis Directs Tissue Restoration via Amphiregulin-Induced Transforming Growth Factor Beta Activation

**DOI:** 10.1016/j.immuni.2019.01.008

**Published:** 2019-03-19

**Authors:** Carlos M. Minutti, Rucha V. Modak, Felicity Macdonald, Fengqi Li, Danielle J. Smyth, David A. Dorward, Natalie Blair, Connor Husovsky, Andrew Muir, Evangelos Giampazolias, Ross Dobie, Rick M. Maizels, Timothy J. Kendall, David W. Griggs, Manfred Kopf, Neil C. Henderson, Dietmar M. Zaiss

**Affiliations:** 1 Institute of Immunology and Infection Research, School of Biological Sciences, University of Edinburgh, Edinburgh EH9 3FL, UK; 2 Immunobiology Laboratory, The Francis Crick Institute, 1 Midland Road, London NW1 1AT, UK; 3 Department of Biology, Institute of Molecular Health Sciences, Swiss Federal Institute of Technology Zurich, Zürich 8093, Switzerland; 4 Wellcome Centre for Molecular Parasitology, Institute for Infection, Immunity and Inflammation, University of Glasgow, Glasgow G12 8TA, UK; 5 Centre for Inflammation Research, University of Edinburgh, Edinburgh EH16 4TJ, UK; 6 Division of Pathology, University of Edinburgh, Edinburgh EH4 2XU, UK; 7 Department of Molecular Microbiology and Immunology, Saint Louis University, Edward A. Doisy Research Center, St. Louis, MO 63104, USA

**Keywords:** Macrophages, TGFb, Amphiregulin, pericytes

## Abstract

The epidermal growth factor receptor ligand Amphiregulin has a well-documented role in the restoration of tissue homeostasis after injury; however, the mechanism by which Amphiregulin contributes to wound repair remains unknown. Here we show that Amphiregulin functioned by releasing bioactive transforming growth factor beta (TGF-β) from latent complexes via integrin-α_V_ activation. Using acute injury models in two different tissues, we found that by inducing TGF-β activation on mesenchymal stromal cells (pericytes), Amphiregulin induced their differentiation into myofibroblasts, thereby selectively contributing to the restoration of vascular barrier function within injured tissue. Furthermore, we identified macrophages as a critical source of Amphiregulin, revealing a direct effector mechanism by which these cells contribute to tissue restoration after acute injury. Combined, these observations expose a so far under-appreciated mechanism of how cells of the immune system selectively control the differentiation of tissue progenitor cells during tissue repair and inflammation.

## Highlights

• Macrophages express Amphiregulin upon tissue damage
• Amphiregulin activates integrin-α_V_ complexes on pericytes
• Integrin-α_V_-activated TGF-β induces pericyte into myofibroblast differentiation
• Myofibroblast-derived collagen contributes to wound healing

## Graphical Abstract

**Figure:**
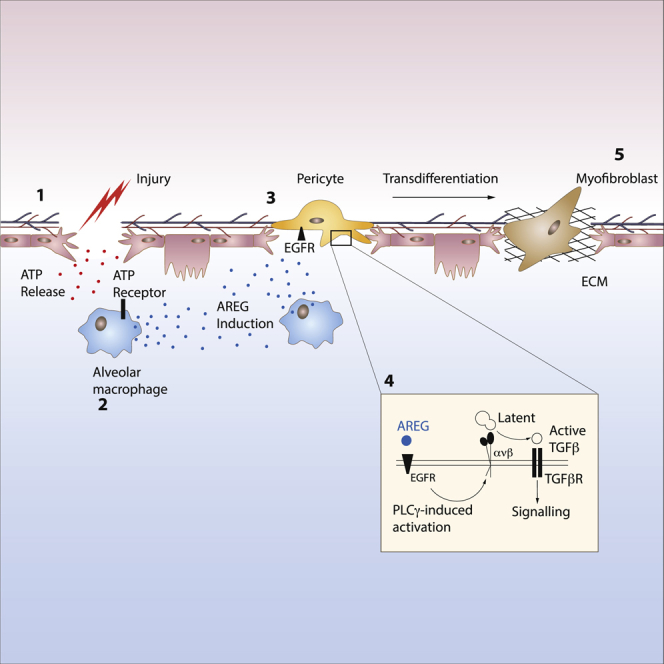

## Teaser

How the immune system uses evolutionarily conserved signaling pathways involved in tissue development to orchestrate wound repair following injury has remained largely unresolved. Here, Minutti et al. show that the macrophage-derived EGFR ligand Amphiregulin induces local TGF-β activation and thereby the differentiation of tissue-resident pericytes.

## Introduction

Maintenance of tissue integrity is a critical process in the development and survival of an organism. Disruption of tissue homeostasis through infections or injury induces a local immune response that facilitates a tissue repair process that in many respects resembles the process of organ development. During this process of wound repair, cells of the immune system support cell proliferation and differentiation in a well-orchestrated manner, ensuring successful tissue regeneration and wound closure (Aurora and Olson, 2014, Martin and Leibovich, 2005, Mescher and Neff, 2005).

Accordingly, the immune system has adapted evolutionarily conserved signaling pathways, such as, for instance, transforming growth factor beta (TGF-β) and the epidermal growth factor receptor (EGFR). These signal transduction pathways play critical roles during both physiological processes: tissue development and wound repair. However, the exact role of these pathways, the cellular triggers, and their interactions during tissue regeneration remain incompletely understood. This is, in part, due to the fact that both pathways have exceptionally pleiotropic functions, but it is also due to the wide variety of different ligands that bind to these two receptors and whose activity is strongly influenced by factors such as the nature of ligand binding to the receptor (Freed et al., 2017), the state of local inflammation, and the state of the receiving cell (Massagué, 2000).

In particular, the EGFR ligand Amphiregulin, expressed under inflammatory conditions by several types of leukocytes, has emerged as a critical player in immunity, inflammation, and tissue repair (Berasain and Avila, 2014, Zaiss et al., 2015). In numerous experimental settings, the delivery of recombinant Amphiregulin (rAREG) enhances the process of tissue repair after injury (Arpaia et al., 2015, Burzyn et al., 2013, Jamieson et al., 2013, Monticelli et al., 2011). Nevertheless, the underlying mechanism that underpins the contribution of Amphiregulin to tissue repair and how it interacts with other known mediators of this process to facilitate wound healing remain largely unexplored.

We therefore sought to determine the mechanism by which Amphiregulin contributes to the restoration of tissue homeostasis after acute tissue injury. Thereby, we have uncovered a mechanism by which EGFR-mediated signaling regulates local TGF-β activity. We found that Amphiregulin expression induced the integrin-α_V_-mediated conversion of latent TGF-β into its bioactive form and, in turn, promoted the differentiation of tissue progenitor cells. After acute tissue injury, this mechanism enabled tissue-resident macrophage-derived Amphiregulin to induce TGF-β activation and the differentiation of pericytes into collagen-producing myofibroblasts, thereby causing rapid tissue re-vascularization and wound healing.

## Results

### Amphiregulin Contributes to the Restoration of Blood Vessel Integrity and Lung Function

**Figure 1:**
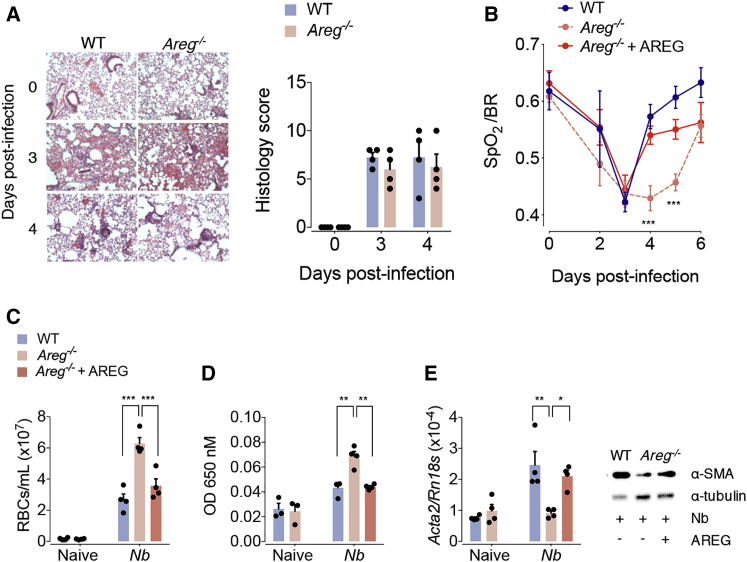
Amphiregulin Contributes to the Restoration of Blood Barrier and Lung Function WT and *Areg*^−/−^ mice were either left uninfected or infected with *N. brasiliensis* and either injected with 5 μg of rAREG at days 1, 2, and 3 post infection or left untreated. (A) Representative H&E staining and histological analysis of lung tissue at different dpi (days post infection). (B) Oxygen saturation in the blood at different dpi. (C) Number of red blood cells in the BAL (bronchoalveolar lavage). (D) Extravasation of Evans blue into the alveolar space as a marker of vascular permeability. (E) mRNA (*Acta2*) and protein expression of the αSMA at 4 dpi were evaluated by qRT-PCR and WB. All data are representative of at least two independent experiments (mean ± SEM); results for individual mice are shown as dots. See also Figure S1.

To determine the physiological relevance of endogenously expressed Amphiregulin during acute wound healing, we utilized a model of acute lung injury caused by infection with the nematode *Nippostrongylus brasiliensis* (Chen et al., 2012, Minutti et al., 2017b, Sutherland et al., 2014). After inoculation, *N. brasiliensis* larvae migrate through the lungs, causing damage to the epithelium and vasculature, which leads to loss of lung function and a drop in blood oxygen saturation (Nieves et al., 2016). After *N. brasiliensis* infection, *Areg*^−/−^ and C57BL/6 wild-type (WT) mice showed a similar extent of lung damage (Figure 1A) and loss of lung function (Figure 1B). Also, the influx of leukocytes into the lungs was similar in composition and number (Figure S1A), and the migration of *Nippostrongylus* larvae through the lungs into the intestine was not affected by Amphiregulin deficiency (Figure S1B). However, in the recovery phase, *Areg*^−/−^ mice presented a significantly delayed restoration of lung function in comparison to WT mice (Figure 1B). This delay in recovery was associated with a diminished restoration of blood vessel integrity as measured by the number of red blood cells and the extravasation of Evans blue dye in the bronchoalveolar lavage (Figures 1C and 1D). Furthermore, *Areg*^−/−^ mice had a diminished transcriptional expression of collagen 1α types I and III (Figure S1C) and αSMA, a marker of myofibroblast differentiation (Figure 1E), on day 4 post infection. Importantly, all the features of Amphiregulin deficiency could be fully reversed by injection of recombinant Amphiregulin (rAREG) (Figures 1B–1E).

Similarly, in a model of acute liver damage induced by injection of the hepatotoxin carbon tetrachloride (CCl_4_), *Areg*^−/−^ and C57BL/6 WT mice showed a similar severity of liver damage and overall recovery after CCl_4_ injection (Figures S1D and S1E); however, *Areg*^−/−^ mice showed a significantly delayed restoration of blood barrier function in the liver, similar to results in the lungs (Figure S1F).

These data suggest that, after acute tissue damage, Amphiregulin mainly contributes to the process of wound healing by enhancing the restoration of vasculature barrier function.

### Macrophage-Derived Amphiregulin Contributes to the Restoration of Vascular Integrity

To investigate the physiologically relevant cellular source of Amphiregulin after *N. brasiliensis* infection, we first established a mouse strain with an Amphiregulin deficiency specifically within hematopoietic cells (*Vav1-cre* × *Areg*^fl/fl^). By infecting this strain with *N. brasiliensis* larvae, we found a substantially delayed recovery of lung and blood barrier function, suggesting that the main source of Amphiregulin contributing to the restoration of the blood barrier function must be of hematopoietic origin (Figures S1G–S1K). Because T cells have been shown to produce Amphiregulin (Arpaia et al., 2015, Burzyn et al., 2013, Zaiss et al., 2006), we assessed lung repair after *N. brasiliensis* infection in mice that lack T and B cells (*Rag1*^−/−^). Because the absence of an adaptive immune system did not influence the extent of blood extravasation in *Rag1*^−/−^ mice compared to that of WT mice (Figure S1L), we conclude that mainly innate immune cells produce Amphiregulin that contributes to the process of wound healing after *N. brasiliensis* infection.

**Figure 2:**
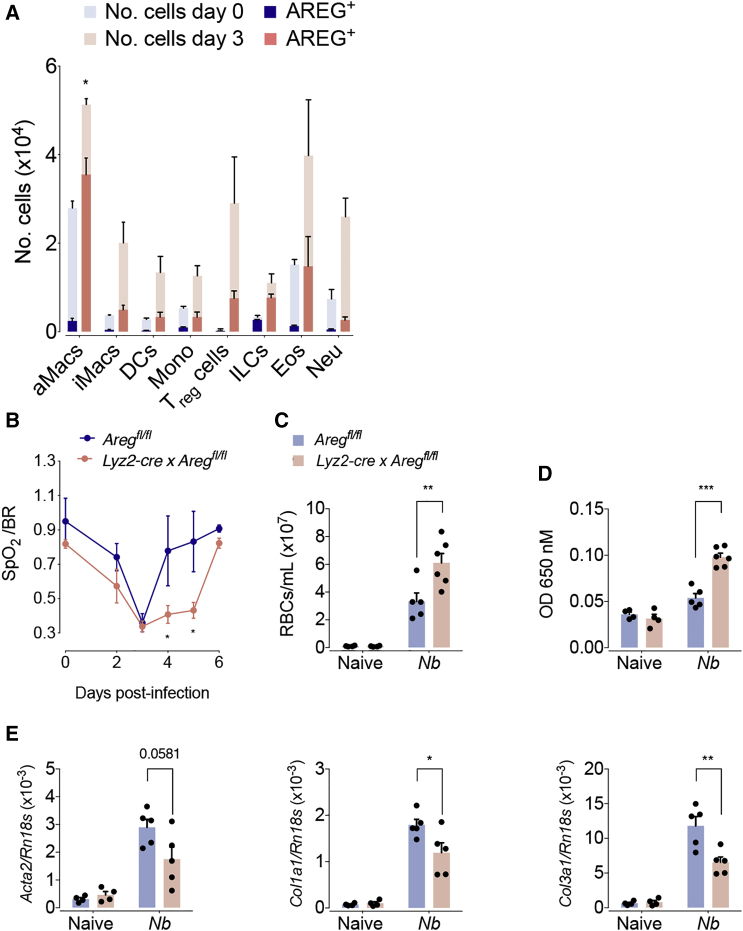
Macrophage-Derived Amphiregulin Contributes to the Restoration of Blood Barrier Function WT and *Areg*^flox/flox^ × *Lyz2cre* mice were either left uninfected or infected with *N. brasiliensis*. (A) Absolute number of Amphiregulin expressing leukocytes following brefeldin A injection and intracellular cytokine staining of whole lung lysates at 3 dpi. (B) Oxygen saturation in the blood at different dpi. (C) Number of red blood cells in the BAL. (D) Extravasation of Evans blue into the alveolar space. (E) Expression of αSMA and collagen α1 type-I- and type-III-encoding genes at 4 dpi were evaluated. All data are representative of at least two independent experiments (mean ± SEM); results for individual mice are shown as dots. See also Figure S2.

To investigate the innate cell population that produces Amphiregulin after tissue injury in more detail, we injected brefeldin A on day 3 after *N. brasiliensis* infection. Injection of brefeldin A prevents protein secretion; thus, subsequent Amphiregulin staining in lung cell suspensions allowed us to reliably detect Amphiregulin expression by different hematopoietic cell types *in vivo* (Figure S2A). Although we detected Amphiregulin expression by several types of innate cells, the induction of Amphiregulin expression was most pronounced in alveolar macrophages (Figures 2A, S2B, and S2C), which were also one of the most frequent types of leukocytes appearing in the lungs over the first three days of infection (Figure S2B). Thus, although regulatory T (Treg) cells, eosinophils, and ILCs also produced considerable amounts of Amphiregulin (Figure S2C), macrophages appeared to be a major source of Amphiregulin in infected lungs (Figures 2A, S2B, and S2C). We therefore generated a mouse strain with a myeloid-specific deficiency of Amphiregulin (*Lyz2cre* × *Areg*^fl/fl^), which showed an alveolar-macrophage-specific lack of Amphiregulin expression (Figure S2D). After *N. brasiliensis* infection, *Lyz2cre* × *Areg*^fl/fl^ mice showed a similar worm burden and an increase in inflammatory infiltrates indistinguishable from that seen in WT mice (Figures S2E and S2F). However, in comparison to WT mice, the genetically modified mouse strain showed an impaired restoration of lung function and blood vessel integrity (Figures 2B–2D) after *N. brasiliensis* infection and impaired induction of collagen 1α type I and type III and αSMA expression (Figure 2E).

To ensure that Amphiregulin deficiency does not impair the functionality of alveolar macrophages during lung repair, we tested the ability of alveolar macrophages from *Lyz2cre* × *Areg*^fl/fl^ mice to acquire an alternative activation program and to proliferate after *N. brasiliensis* infection. Because we could not find any substantial differences between Amphiregulin-deficient and WT macrophage proliferation and differentiation (Figure S2G), we concluded that Amphiregulin is not contributing to these processes.

Moreover, comparable to our observations in the lungs, *Lyz2cre* × *Areg*^fl/fl^ mice showed, in comparison to WT littermates, a delayed restoration of blood vessel integrity after CCl_4_-induced liver damage, despite experiencing similar severity of liver damage and overall recovery after CCl_4_ injection (Figures S2H–S2J).

These data suggest that macrophage-derived Amphiregulin has no effect on alveolar macrophages themselves but directly contributes to wound repair by enhancing the restoration of blood vessel integrity after *N. brasiliensis* infection.

### Extracellular ATP Is an Important Stimulus Inducing Amphiregulin Expression in Macrophages

**Figure 3:**
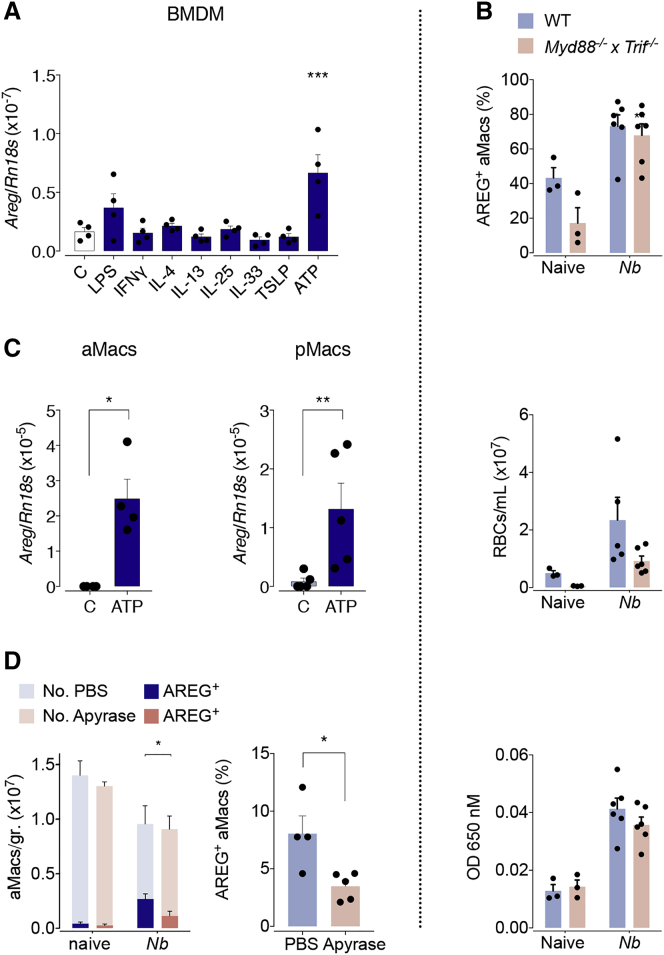
Sensing Extracellular ATP from Tissue Necrosis Drives Amphiregulin Expression by Macrophages (A) *In vitro* differentiated bone marrow derived macrophages were treated as indicated. Expression of Amphiregulin-encoding gene was measured 10 h after treatment. (B) WT and *Myd88*^*−/−*^ × *Trif*^*−/−*^ mice were either left uninfected or infected with *N. brasiliensis*. Percentage of Amphiregulin-expressing (cell surface and intracellular after i.v. injection of brefeldin A) macrophages at 3 dpi (upper panel), number of red blood cells in the BAL (middle panel), and extravasation of Evans blue into the alveolar space at 4 dpi (lower panel). (C) Alveolar and peritoneal macrophages were purified by adherence and then treated with ATP. Expression of Amphiregulin-encoding gene was measured 10 h after treatment. (D) WT mice were either left uninfected or infected with *N. brasiliensis* and either received two individual doses of Apyrase at day 1 post-infection, or did not. Number and percentage of Amphiregulin expressing alveolar macrophages following brefeldin A injection and intracellular cytokine staining of lung lysate at 2 dpi. All data are representative of at least two independent experiments (mean ± SEM); results for individual mice are shown as dots. See also Figure S3.

Having identified alveolar macrophages as critical sources of Amphiregulin at the initiation of tissue repair after acute lung injury, we wanted to identify the factors that induce its expression. Previous studies have shown that several damage-associated molecular patterns (DAMPs), such as alarmins (interleukin [IL]-33, IL-25, and TSLP [thymic stromal lymphopoietin]) or extracellular ATP, can induce Amphiregulin expression in leukocytes (Zaiss et al., 2015). Therefore, to test their capacity to induce Amphiregulin expression in macrophages, we differentiated bone-marrow-derived macrophages (BMDMs) *in vitro* and measured *Areg* mRNA expression upon treatment with these molecules. We also treated BMDMs with factors that induce classical (lipopolysaccharide [LPS] and interferon [IFN]-γ) and alternative (IL-4 and IL-13) activation of macrophages to test if Amphiregulin expression by macrophages was associated with their activation. As shown in Figure 3A, we observed that mainly ATP and LPS, but not IL-33, induced Amphiregulin expression. To confirm these findings *in vivo*, we measured Amphiregulin expression and the process of lung repair after *N. brasiliensis* infection in WT and *Myd88*^−/−^ × *Trif*^−/−^ mice, a mouse strain lacking the ability to propagate the signaling caused by LPS and IL-33. In line with the fact that the overall influx of leukocytes into the lungs of infected mice was not affected (Figure S3A), the induction of Amphiregulin expression by macrophages after *N. brasiliensis* infection was also not impaired in *Myd88*^−/−^ × *Trif*^−/−^ mice (Figure 3B). Accordingly, *Myd88*^−/−^ × *Trif*^−/−^ mice recovered the vascular barrier function in a comparable way to WT mice 4 days after infection (Figure 3B). These data strongly suggest that neither LPS nor IL-33 is involved in the induction of Amphiregulin expression by alveolar macrophages.

To determine the capacity of extracellular ATP to induce Amphiregulin expression in tissue-resident macrophages, we isolated macrophages from the alveolar space and the peritoneal cavity and exposed them to ATP. Similar to BMDMs, peritoneal and alveolar macrophages readily induced Amphiregulin expression upon ATP treatment (Figure 3C).

Finally, to test the impact of ATP sensing by alveolar macrophages during *N. brasiliensis* infection, we depleted extracellular ATP by treating WT mice with Apyrase at day 1 post infection, when the infective larvae start colonizing the lungs. Although the overall influx of leukocytes into the lungs of infected mice and the migration of worm larvae through the lungs were not affected (Figures S3B and S3C), we observed that a day after Apyrase delivery, Amphiregulin expression by alveolar macrophages was significantly diminished (Figure 3D).

These findings strongly suggest that sensing extracellular ATP allows alveolar macrophages to recognize tissue damage and induce the expression of Amphiregulin, allowing for a rapid repair of the vascular barrier function.

### Amphiregulin Induces the Activation of Integrin-α_V_ and Consequently the Release of Bioactive TGF-β

**Figure 4:**
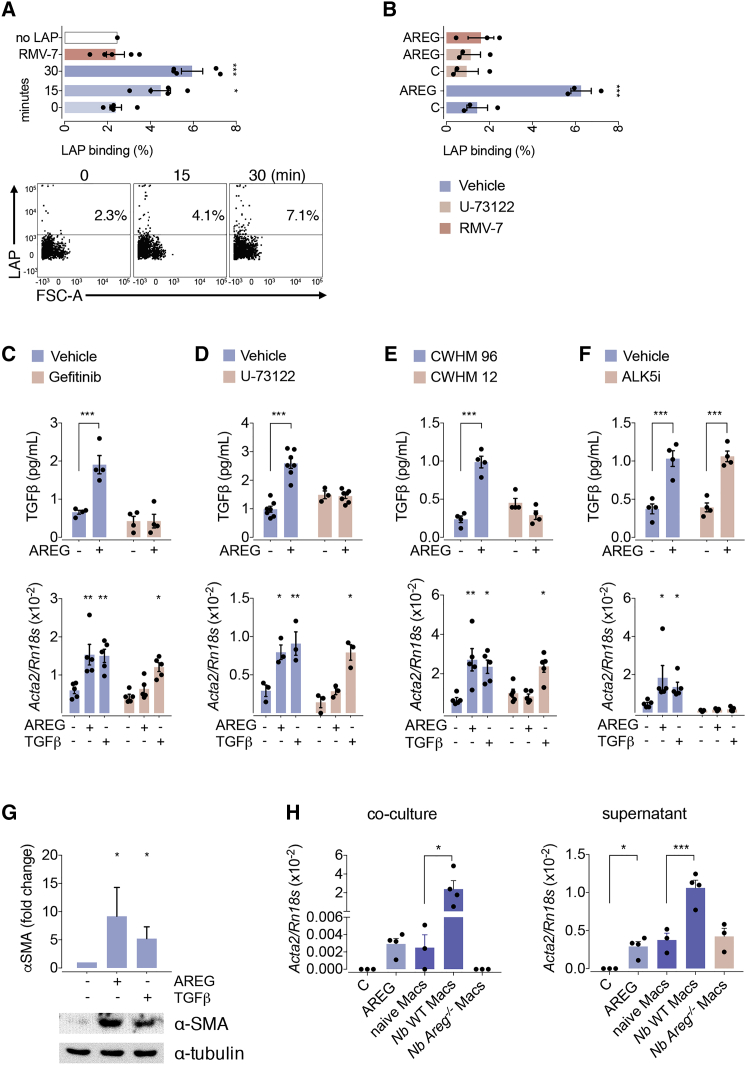
Amphiregulin Induces Pericyte Differentiation by Releasing Bioactive TGF-β via Integrin-α_V_ (A and B) Primary lung pericytes were cultured in the presence or absence of 100 ng/ml Amphiregulin (A), then incubated with latent TGF-β in the presence and absence of PLCγ inhibitor U-73122 (B) and analyzed for LAP binding by flow cytometry. (C–F) After 24 h of treatment, the release of bioactive TGF-β (upper panel), as well as their differentiation into myofibroblasts (lower panels), was determined in the presence or absence of inhibitors for the EGFR (Gefitinib) (C), PLCγ inhibitor (U-73122) (D), integrin-α_V_ (CWHM-12 and its inactive control enantiomer CWHM-96) (E), or TGF-β-R (ALK5i) (F). (G) The induction of αSMA in treated pericytes was also evaluated at the protein level by western blot analysis. (H) Differentiation of primary lung pericytes into myofibroblasts following o/n co-culture with (left graph) or o/n exposure to supernatants derived from (right graph) alveolar macrophages isolated from infected or uninfected WT or *Areg^-/-^* mice. All data are representative of at least two independent experiments except for (B)–(D) (mean ± SEM); results for preparations from individual mice are shown as dots. See also Figure S4.

Having established the source of Amphiregulin, we wanted to know the downstream effector mechanisms that underpin Amphiregulin’s effects on vascular repair. Mesenchymal stromal cells, or so-called pericytes, are a major myofibroblast precursor cell type in the lungs and liver (Henderson et al., 2013) known to promote integrity of blood vessels (Lindahl et al., 1997). Myofibroblast differentiation under inflammatory conditions is TGF-β driven (Henderson et al., 2013). TGF-β is secreted in the form of a latent, inactive protein complex and is locally released into its bioactive form, for instance, in an integrin-α_V_-mediated step (Gleizes et al., 1997, Henderson and Sheppard, 2013, Munger et al., 1999). Because the delayed restoration of lung function in *Areg*^−/−^ mice was directly associated with a diminished expression of the myofibroblast differentiation marker αSMA (*Acta2*) (Figure 1E), we hypothesized that Amphiregulin may induce the release of bioactive TGF-β by activating integrin-α_V_-containing integrin complexes. To test this hypothesis, we isolated primary platelet-derived growth factor receptor-β (PDGFRβ)-expressing pericytes and exposed them to latent TGF-β in the presence and absence of rAREG. We found that although rAREG treatment did not increase the transcription or the cell-surface expression of integrin-α_V_ on cultured pericytes (Figures S4A and S4B), it enhanced the binding of the TGF-β latent associated protein (LAP) to pericytes (Figure 4A), a process that could be fully reverted by the addition of an integrin-α_V_-blocking antibody RMV-7 (Figure 4A). These data suggest that rAREG induced the activation of integrin-α_V_-containing integrin complexes on pericytes.

The activation of integrin-α_V_ complexes by Amphiregulin recapitulated all the hallmarks of an “inside-out” activation of integrins triggered by other growth factors (Shattil et al., 2010). In this process, the growth factor induces sustained phospholipase-Cγ (PLCγ) signaling that leads to cytoskeleton rearrangements within the cell, which then pull the two integrin subunits apart and, in this way, open their structure on the cell surface (Shattil et al., 2010). Therefore, to test whether rAREG induces the “inside-out” activation of integrin-α_V_, we repeated the latter experiment in the presence of the PLCγ inhibitor U-73122. As shown in Figure 4B, such treatment fully prevented the binding of LAP to integrin-α_V_, strongly suggesting that Amphiregulin induces the activation of integrin-α_V_ via an “inside-out”-mediated mechanism.

Based on these findings, we further queried whether the rAREG-induced activation of integrin-α_V_ may also induce the release of bioactive TGF-β and in this way induce the differentiation of pericytes into myofibroblasts. In accordance with such a hypothesis, we found that, although rAREG did not influence the secretion of total TGF-β from primary pericytes (Figure S4C), it induced the release of bioactive TGF-β from its latent form (upper panel, Figures 4C–4F; Figure S4D). The release of bioactive TGF-β was prevented by the addition of the EGFR inhibitor Gefitinib (Figure 4C) and was absent in cultures of primary pericytes derived from *Pdgfrb-cre* × *Egfr*^fl/fl^ mice (Figure S4D), a mouse strain with a pericyte-specific deletion of the EGFR (Henderson et al., 2013). Furthermore, the blockade of PLCγ signaling (Figure 4D, upper panel) or of integrin-α_V_ by CWHM-12 (Henderson et al., 2013) (Figure 4E, upper panel), but not of TGF-β-RI by an ALK5 inhibitor (Figure 4F, upper panel), blocked the release of bioactive TGF-β by rAREG-treated pericytes.

In accordance with rAREG release of bioactive TGF-β, we found that the addition of rAREG also induced the differentiation of pericytes into myofibroblasts (lower panel, Figures 4C–4F; Figure S4D), which was an effect fully reverted by the addition of the EGFR inhibitor Gefitinib (Figure 4C), the PLCγ signaling inhibitor U-73122 (Figure 4D), the integrin-α_V_ inhibitor CWHM-12 (Figure 4E, lower panel), or TGF-β-RI inhibition (Figure 4F, lower panel) and which was absent in cultures of pericytes derived from *Pdgfrb-cre* × *Egfr*^fl/fl^ mice (Figure S4D). Importantly, pericytes from *Pdgfrb-cre* × *Egfr*^fl/fl^ mice remained responsive to TGF-β (Figure S4D), further confirming our observations using pharmacological inhibitors of EGFR.

Furthermore, to demonstrate that the quantification of *Acta2* mRNA was a bona fide surrogate measurement of the differentiation of pericytes into myofibroblasts, we performed immunoblot analysis of treated pericytes with anti-αSMA specific antisera. As shown in Figure 4G, the expression of αSMA in isolated pericytes significantly increased upon exposure to rAREG or recombinant (r)TGF-β, formally demonstrating that Amphiregulin induces the differentiation of pericytes into myofibroblasts.

Finally, in order to formally demonstrate that macrophage-derived Amphiregulin can also induce the differentiation of pericytes, we isolated alveolar macrophages from *N. brasiliensis*-infected mice (WT and *Areg*^−/−^) and co-cultured them with primary pericytes. As shown in Figure 4H, the co-culture of alveolar macrophages derived from WT mice*,* but not from *Areg*^−/−^ mice, induced the differentiation of pericytes into myofibroblasts. Also, the supernatants of overnight-cultured alveolar macrophages from infected WT mice, but not from *Areg*^−/−^ mice, induced the differentiation of pericytes into myofibroblasts.

Combined, these data reveal a mechanism by which Amphiregulin induces the activation of integrin-α_V_ on pericytes, causing the local release of bioactive TGF-β from latent TGF-β and thus their differentiation into myofibroblasts.

### rTGF-β Reverts the Effects of Amphiregulin and Pericyte-Specific EGFR Deficiency *In Vivo*

**Figure 5:**
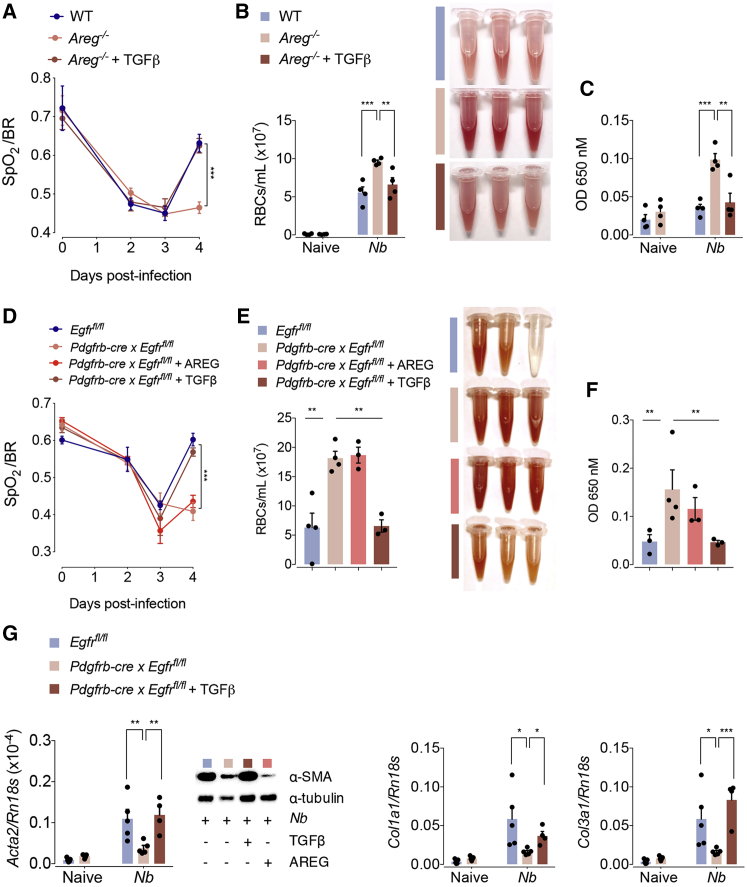
r TGF-β Restores Tissue Repair in Areg^−/−^ Mice (A–C) WT or *Areg*^*−/−*^ mice were either left uninfected or infected with *N. brasiliensis.* On days 1, 2, and 3 pi, mice were treated with 5 μg of rTGFβ or left untreated. (A) Oxygen saturation in the blood at different dpi, (B) number of red blood cells in the BAL, and (C) extravasation of Evans blue into the alveolar space at 4 dpi were evaluated. (D–G) *Egfr*^flox/flox^ or *Egfr*^flox/flox^ × *Pdgfrb-cre* mice were either left uninfected or infected with *N. brasiliensis.* On days 1, 2, and 3 pi, mice were treated with 5 μg of either rAREG or rTGFβ or left untreated. (D) Oxygen saturation in the blood at different dpi, (E) number of red blood cells in the BAL, (F) extravasation of Evans blue into the alveolar space, and (G) expression of the αSMA and collagen α1 types I and III were evaluated at 4 dpi. Data represent mean ± SEM; results for individual mice are shown as dots. See also Figure S5.

Next, we sought to reveal the physiological relevance of the mechanism we had found *in vitro*. In accordance with our previous observation that Amphiregulin mediates TGF-β activation, we found that *N. brasiliensis*-infected *Areg*^−/−^ mice show, in comparison to WT C57BL/6 counterparts, a diminished expression of pSMAD3, the main mediator of TGF-β signaling (Figure S4E). This suggests that Amphiregulin *in vivo* also contributes to the release of bioactive TGF-β. To test the link between Amphiregulin and TGF-β, we injected rTGF-β into *N. brasiliensis*-infected *Areg*^−/−^ mice. As shown in Figures 5A–5C, the injection of rTGF-β fully reverted the deficiency of *Areg*^−/−^ mice in the restoration of lung function and blood vessel integrity.

To dissect this mechanism further, we analyzed *Pdgfrb-cre* × *Egfr*^fl/fl^ mice, a mouse strain with a pericyte-specific deficiency of EGFR expression. At steady state, *Pdgfrb-cre* × *Egfr*^fl/fl^ mice showed no reduced transcriptional expression of *Pdgfrb* in comparison to WT controls, and thus we conclude that EGFR ablation on pericytes did not affect the development or survival of this cell population (Figure S5A). Nevertheless, when mice were infected with *N. brasiliensis*, we found that, similar to *Areg*^−/−^ mice, *Pdgfrb-cre* × *Egfr*^fl/fl^ mice also showed a diminished restoration of lung function (Figure 5D) and blood vasculature integrity (Figures 5E and 5F). Also, αSMA and collagen gene expression on day 4 post infection was diminished in comparison to WT mice (Figure 5G). *Pdgfrb-cre* × *Egfr*^fl/fl^ mice showed a similar influx of leukocytes into the lungs (Figure S5B), a comparable alveolar damage (Figure S5C), and a similar worm burden (Figure S5D) to WT mice. Furthermore, similar results were found in the liver, with the restoration of blood barrier function being selectively delayed after CCl_4_-induced damage in *Pdgfrb-cre* × *Egfr*^fl/fl^ mice compared to WT littermates, despite a similar extent of inflammation and necrosis (Figures S5E–S5H).

Moreover, although the injection of rTGF-β into *N. brasiliensis*-infected *Pdgfrb-cre* × *Egfr*^fl/fl^ mice (Figures 5D–5G) fully restored their lung function, blood barrier integrity, and myofibroblast differentiation on day 4 post infection, the administration of rAREG did not revert this phenotype in *Pdgfrb-cre* × *Egfr*^fl/fl^ mice (Figures 5D–5F).

Taken together, these data demonstrate that Amphiregulin functions upstream of TGF-β in the differentiation of pericytes into myofibroblasts during acute tissue damage.

### Amphiregulin-Induced Integrin-α_V_ Activation Promotes Vascular Repair

**Figure 6:**
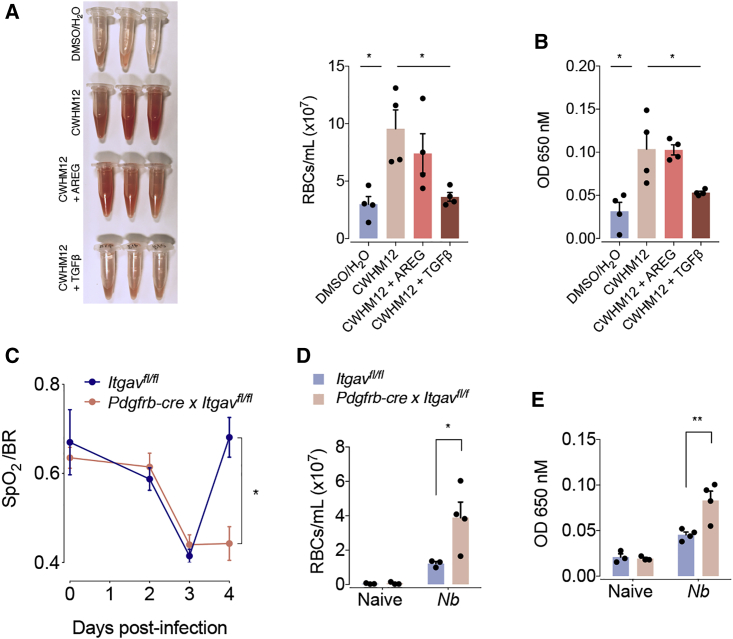
Amphiregulin Restores Blood Barrier Function via Pericyte-Specific Activation of Integrin-α_V_ Complexes (A and B) WT mice were infected with *N. brasiliensis* or left uninfected, and minipumps containing the integrin-α_V_ inhibitor CWHM12 were inserted subcutaneously 3 days prior to infection. Mice were treated with 5 μg of either rAREG or rTGFβ 1, 2, and 3 dpi. (A) Number of red blood cells in the BAL and (B) extravasation of Evans blue into the alveolar space were evaluated 4 dpi. (C–E) *Igtav*^flox/flox^ and *Igtav*^flox/flox^ × *Pdgfrb-cre* mice were infected with *N. brasiliensis* or left uninfected. (C) Oxygen saturation in the blood at different dpi. (D) Number of red blood cells in the BAL and (E) extravasation of Evans blue into the alveolar space were evaluated 4 dpi. Data represent mean ± SEM; results for individual mice are shown as dots. See also Figure S6.

To address the role of Amphiregulin-induced integrin-α_V_ activation in the restoration of lung function during *N. brasiliensis* infection, we inserted mini pumps containing the integrin-α_V_ inhibitor CWHM-12 into C57BL/6 WT mice prior to *N. brasiliensis* infection. We found that treatment with the integrin-α_V_ inhibitor CWHM12 had no effect on the increase in inflammatory infiltrates into the lungs or the migration of worms into the intestine (Figures S6A and S6B). Nevertheless, mice treated with the integrin-α_V_ inhibitor CWHM12 showed a diminished restoration of blood barrier function after *N. brasiliensis* infection (Figures 6A and 6B). This effect could fully be reverted by the administration of rTGF-β but not by rAREG (Figures 6A and 6B). These data clearly demonstrate that Amphiregulin effects occur upstream of integrin-α_V_ activation.

Finally, to directly demonstrate the involvement of integrin-α_V_ on pericytes during vascular repair, we generated pericyte-specific integrin-α_V_-deficient mice (*Pdgfrb-cre* × *Itgav*^fl/fl^) and infected these with *N. brasiliensis* larvae. As shown in Figures 6C–6E, pericyte-specific deficiency of integrin-α_V_ directly reproduced the effects of systemic CWHM-12 delivery during *N. brasiliensis* infection—again showing a delay in the recovery of lung function and vascular repair, despite having a similar influx of leukocytes into the lungs and worm migration into the intestine (Figures S6C and S6D).

These experiments demonstrate that *in vivo* Amphiregulin also contributes to the restoration of tissue integrity by inducing integrin-α_V_-mediated TGF-β release specifically on pericytes.

## Discussion

Our data reveal a mechanism by which Amphiregulin induces the activation of integrin-α_V_ complexes on pericytes and, in this way, induces the local release of bioactive TGF-β. This in turn induces their differentiation into collagen-producing myofibroblasts, which critically contributes to the restoration of vascular integrity in injured tissues.

This link between Amphiregulin-induced EGFR signaling and TGF-β activation may explain, in addition to its role in wound healing, several formerly described effects associated with Amphiregulin expression. Amphiregulin has been associated with tissue fibrosis (Perugorria et al., 2008), regulatory T-cell-mediated immune regulation (Zaiss et al., 2013), and tumor growth (Khambata-Ford et al., 2007, Li et al., 2010, Tinhofer et al., 2011). As all of these processes are strongly influenced by TGF-β-induced signaling (Li et al., 2006), our data suggest that, in these situations also, Amphiregulin may function by inducing the activation of locally expressed latent TGF-β. To target TGF-β directly in a therapeutic setting is currently strongly hindered by its housekeeping function, for instance, due to its role in heart muscle homeostasis. However, TGF-β can be activated in different ways, and the inflammatory activation is mainly mediated by integrin-α_V_ activation (Henderson and Sheppard, 2013). Therefore, our finding that Amphiregulin regulates TGF-β function under inflammatory conditions may propose targeting Amphiregulin activity as an attractive alternative therapeutic approach in the context of tumor therapy or chronic inflammation-associated fibrotic diseases.

Our findings furthermore elaborate a previously unrecognized way by which macrophages contribute to wound healing. It is well-established that macrophages contribute to the process of wound healing by removing cell debris and the release of growth factors that, for example, stimulate angiogenesis or fibroblast replication and subsequently dampen inflammation (Minutti et al., 2017c, Wynn et al., 2013). Our data now show that by responding to the breach of barrier function and controlling the local release of bioactive TGF-β, macrophages also control the differentiation of local tissue precursor cells. This finding dovetails with previous studies demonstrating that alveolar macrophages in particular play an essential role in limiting acute tissue damage during experimental helminth infection (Chen et al., 2012, Minutti et al., 2017b) and reveals an additional function for a cell type, optimally located to function as a sentinel of tissue injury.

This specific location of macrophages may also explain the specific role macrophage-derived Amphiregulin plays during the restoration of blood vessel integrity. Several types of leukocytes have previously been described to express Amphiregulin and to contribute to wound healing (Arpaia et al., 2015, Burzyn et al., 2013, Jamieson et al., 2013, Monticelli et al., 2011); however, in our case, macrophage-derived Amphiregulin specifically appears to play a central role. One potential explanation could be based on the fact that, soon after injury, macrophages are drawn to wound blood vessels (Gurevich et al., 2018). In this way, macrophages are optimally situated to sense the release of ATP from necrotic cells and to locally produce physiologically critical amounts of Amphiregulin, which then induce the local activation of bioactive TGF-β and thus the differentiation of blood-vessel-associated pericytes. In this way, the locally expressed, macrophage-derived Amphiregulin can critically contribute to wound healing, whereas Amphiregulin produced by other leukocytes at other sites within the lungs might be of less importance for the restoration of blood vessel integrity.

Furthermore, the cross-talk between EGFR and TGF-β described here substantially contributes to our understanding of how the function of the pleiotropic cytokine TGF-β is regulated. A cross-talk between EGFR and TGF-β signaling has been proposed before. However, so far these studies have focused exclusively on the high-affinity EGFR ligand EGF, which interferes with TGF-β intra-cellular signaling and counteracts its functioning (Lo et al., 2001, Massagué, 2000), thereby inducing the proliferation and preventing the differentiation of tissue stem cells (Basak et al., 2017). In contrast to EGF, Amphiregulin is a low-affinity EGFR ligand (Berasain and Avila, 2014) and as such induces a tonic signal via the EGFR (Freed et al., 2017). Accordingly, Amphiregulin does not induce receptor internalization (Stern et al., 2008) but preferentially induces PLCγ signaling via the phosphorylation of Tyr992 (Gilmore et al., 2008, Minutti et al., 2017a). It is well established that the induction of tonic PLCγ signaling by growth factors induces integrin complex activation (Shattil et al., 2010), which in turn is known to be a critical step in the local conversion of latent into bioactive TGF-β (Robertson and Rifkin, 2016). Thus, in conclusion, our data reveal that EGFR-mediated signaling is a key determinant of the local functionality of TGF-β. Depending on the quality of the EGFR-induced signals, different ligands can either activate TGF-β (Amphiregulin induced) or block its activity (EGF induced).

Such a way to regulate TGF-β function may also resolve the long-standing paradigm that signaling via some receptor-tyrosine kinases, such as NGF via the NGFR, can induce the differentiation of the neural progenitor cells PC-12, whereas other signals, such as EGF via the EGFR, can actively block their differentiation (Traverse et al., 1992). So far, it has been assumed that the qualitative difference in MAP-kinase activation (tonic versus oscillating) is the main driver of these different physiological outcomes (Marshall, 1995). However, our data now suggest a possible alternative explanation for this phenomenon, i.e., that different growth factors induce distinct intra-cellular signals that interfere with the TGF-β signaling pathway (Lo et al., 2001) and thereby either prevent differentiation (Basak et al., 2017) or actively induce the activation of TGF-β, in this way inducing the differentiation of PC-12. Further analysis of the effect of NGF on integrin-α_V_ activation and consecutive release of bioactive TGF-β upon exposure to PC-12 cells may help to confirm such a hypothesis.

The cross-talk between the EGFR and TGF-β unraveled in this manuscript may also explain the selective expression of the EGFR on tissue progenitor cells. This is by far the best studied in the situation of Lgr5-expressing intestinal stem cells. Lgr5-expressing intestinal stem cells have a high expression of the EGFR, which supports their proliferation and prevents their differentiation (Basak et al., 2017). Resting, so-called “reserve” tissue stem cells, however, are physically removed from the proliferating stem cell compartment; they have low expression of the EGFR and even express the molecular EGFR inhibitor LRIG (leucine-rich repeats and immunoglobulin-like domains) (Powell et al., 2012, Wong et al., 2012). Such active inhibition of the EGFR may function to prevent the proliferation of these resting stem cells or, alternatively, to prevent untimely TGF-β-induced differentiation. Under inflammatory conditions, however, the expression of the EGFR on activated tissue stem cells may open a window of opportunity for cells of the immune system to influence the fate decision of tissue stem cells by secreting Amphiregulin, inducing their differentiation. This mechanism could then enable inflamed tissue to rapidly adjust to changes in the state of inflammation, in this way contributing to the restoration and maintenance of tissue integrity.

## STAR★Methods

### Key Resources Table

**Table:** 

REAGENT or RESOURCE	SOURCE	IDENTIFIER
**Antibodies**		

Rat anti mouse CD3 (clone 17A2)	Biolegend	100228
Armenian Hamster anti mouse CD3 (clone 145-2C11)	BD Biosciences	564378
Rat anti-mouse CD16/32	Biolegend	101302
Rat anti mouse CD19 (clone 6D5)	Biolegend	115538
Rat anti mouse CD19 (clone eBio1D3)	eBioscience	17-0193-82
Rat anti mouse SiglecF (clone E50-2440)	BD PharMingen	562681
Rat anti mouse SiglecF (clone E50-2440)	BD PharMingen	552126
Rat anti mouse Ly6G (clone 1A8)	Biolegend	127628
Rat anti mouse Ly6G (clone 1A8)	Biolegend	127624
Rat anti mouse Ly6C (clone HK1.4)	Biolegend	128024
Mouse anti mouse NK-1.1 (clone PK136)	Biolegend	108731
Armenian Hamster anti mouse CD11c (clone N418)	Biolegend	117334
Rat anti CD11b (clone M1/70)	Biolegend	101241
Rat anti mouse CD4 (clone RM4-5)	Biolegend	100536
Rat anti mouse CD4 (clone GK1.5)	eBioscience	11-0041-82
Rat anti mouse F4/80 (clone BM8)	PEproTech Inc.	25-4801-82
Mouse anti mouse CD45.2 (clone 104)	eBioscience	12-0454-81
Mouse anti mouse CD45.2 (clone 104)	Biolegend	109826
Rat anti mouse MHCII (I-A/I-E) (clone M5/114.15.2)	Biolegend	107624
Rat anti mouse MHCII (I-A/I-E) (clone M5/114.15.2)	eBioscience	47-5321-80
Rat anti mouse MHCII (I-A/I-E) (clone M5/114.15.2)	eBioscience	11-5321-82
Rat anti mouse CD90.2 (clone 30-H12)	Biolegend	105327
Rat anti mouse c-kit (clone 2B8)	Biolegend	105813
Hamster anti mouse FcεRIα (clone MAR-1)	Biolegend	134309
Hamster anti mouse CD49b (clone HM ALPHA2)	BD PharMingen	558759
Rat anti mouse ICAM-2 (clone 3C4 MIC2/4)	Biolegend	105612
Rat anti mouse LAP (clone TW7-16B4)	Biolegend	141405
Goat anti mouse amphiregulin (polyclonal)	R&D Systems	BAF989
Rat anti mouse FoxP3 (clone MF23)	BD Biosciences	562466
Rabbit anti mouse RELMα (polyclonal)	PeproTech Inc.	500-P214
Mouse anti human Ki67 (clone B56)	BD Biosciences	556026
Rat anti-mouse CD51 (αV integrin) (clone RMV-7)	Biolegend	104105
Phospho-Smad3 (Ser423/425) (C25A9) Rabbit mAb	Cell Signaling	9520
α-Smooth Muscle Actin (D4K9N) XP Rabbit mAb	Cell Signaling	19245
α-Tubulin (11H10) Rabbit mAb (HRP Conjugate)	Cell Signaling	9099
β-Tubulin (9F3) Rabbit mAb	Cell Signaling	2128

**Biological Samples**		

Rat-adapted *Nippostrongylus brasiliensis*	Sutherland et al., 2014	N/A

**Chemicals, Peptides, and Recombinant Proteins**		

Collagenase B	Roche	11088815001
Collagenase D	Roche	11088866001
Pronase (Protease from Streptomyces griseus)	Sigma	P5147
DNase 1	Roche	10104159001
Recombinant mouse Amphiregulin	BioLegend	554104
Recombinant Mouse TGF-β1	BioLegend	763104
Recombinant Mouse IFN-γ	PeproTech	315-05
Recombinant Mouse IL-4	PeproTech	214-14
Recombinant Mouse IL-13	Immunotools	12340137
Recombinant Mouse IL-25	BioLegend	587304
Recombinant Mouse IL-33	PeproTech	210-33
Recombinant Mouse TSLP	eBiosciences	34-8498-82
Lipopolysaccharides from Escherichia coli O111:B4	Sigma	L4391
ATP	Cytoskeleton	BSA04-001
Gefitinib	LC laboratories	G-4408
U-73122 PLC inhibitor	Cayman Chemical	70740
CWHM96/12	David W. Griggs lab	N/A
ALK inhibitor SB431542	Abcam	ab120163
Carbon Tetrachloride	Sigma	87031
Brefeldin A	Sigma	B6542
Apyrase	Sigma	A6237
Evans blue	Fluka Chemika	46160

**Critical Commercial Assays**		

LIVE/DEAD Fixable Blue Dead Cell Stain Kit	Thermo Fisher	L23105
LEGENDplex Mouse/Rat Free Active/Total TGF-β1	Biolegend	740490

**Experimental Models: Cell Lines**		

L929	The Francis Crick Institute Cell Services	N/A

**Experimental Models: Organisms/Strains**		

*Areg*^−/−^	Luetteke et al., 1999	N/A
*Myd88*^−/−^ × *Trif*^−/−^	Yamamoto et al., 2003, Adachi et al., 1998	N/A
*Rag1*^−/−^	Mombaerts et al., 1992	N/A
*Areg*^flox/flox^	The European Conditional Mouse Mutagenesis Program	*Areg*^*tm2a(EUCOMM)Hmgu*^
*LysM-cre*^*+*/^	Clausen et al., 1999	N/A
*Vav1-cre*^+/−^	de Boer et al., 2003	N/A
*Egfr*^*flox*/*flox*^	Natarajan et al., 2007	N/A
*Itgav*^*flox*/*flox*^	Foo et al., 2006	N/A
*Pdgfrb-cre*^+/−^	Foo et al., 2006	N/A
*Pdgfrb-BAC-eGFP*	Henderson et al., 2013	N/A

**Oligonucleotides**		

*Acta2*	Applied Biosystems	Mm00725412_s1
*Col1a1*	Applied Biosystems	Mm00801666_g1
*Col3a1*	Applied Biosystems	Mm01254476_m1
*Areg*	Applied Biosystems	Mm00437583_m1
*Pdgfrb*	Applied Biosystems	Mm00435553_m1
*Rn18s*	Applied Biosystems	Mm03928990_g1
*Gapdh*	Applied Biosystems	Mm99999915_g1
*Itgav*	Applied Biosystems	Mm00434486_m1

**Software and Algorithms**		

FlowJo 10	FLOWJO, LLC	https://www.flowjo.com/
Prism 7	GraphPad Software	https://www.graphpad.com/scientific-software/prism/

**Other**		

ALZET osmotic minipumps	Charles River	1007D
The MouseOxTM Pulse-oximeter	Starr Life Sciences	N/A

### Contact for Reagent and Resource Sharing

Further information and requests for resources and reagents should be directed to and will be fulfilled by the Lead Contact, Dietmar Zaiss (dietmar.zaiss@ed.ac.uk).

### Experimental Model and Subject Details

#### Breeding of Experimental Animals

*Areg*^−/−^ (Luetteke et al., 1999, Zaiss et al., 2006), *Areg*^*flox*/*flox*^ (*Areg*^*tm2a(EUCOMM)Hmgu*^ obtained from The European Conditional Mouse Mutagenesis Program), *Vav1-cre*^+/−^ (de Boer et al., 2003), *Lyz2cre*^+/−^ (Clausen et al., 1999), *Pdgfrb-cre*^+/−^ (Foo et al., 2006, Henderson et al., 2013), *Egfr*^*flox*/*flox*^ (Natarajan et al., 2007), *Itgav*^*flox*/*flox*^ (Foo et al., 2006, Henderson et al., 2013), *Pdgfrb-BAC-eGFP* (Henderson et al., 2013) reporter mice, as well as *Myd88*^−/−^ × *Trif-*^−/−^ (Adachi et al., 1998, Yamamoto et al., 2003), and *Rag1*^−/−^ (Mombaerts et al., 1992) were bred and maintained on a C57BL/6 background at the University of Edinburgh under specific-pathogen free conditions. Mice were 8–12 weeks old at the start of the experiments and were housed in individually ventilated cages. Mice were not randomized in cages, but each cage was randomly assigned to a treatment group. Investigators were not blinded to mouse identity during necropsy but during worm counts and histological analysis. Male and female mice were used to perform the experiments. However, in no occasion, we observed an obvious difference between sexes within the parameters analyzed for our experiments. Experiments were performed in accordance with the United Kingdom Animals (Scientific Procedures) Act of 1986. The UK Home Office accredited all researchers for animal handling and experimentation. Dispensation to carry out animal research at the University of Edinburgh was approved by the University of Edinburgh Animal Welfare and Ethical Review Body and granted by the UK government Home Office; as such all research was carried under the project license PPL70/8470. Sample sizes were based on extensive previous experience with *Nippostrongylus brasiliensis* infection model. No individual data points were excluded under any circumstances.

### Method Details

#### Primary Cell Isolation

##### Lung

Mouse lungs were perfused through the left ventricle using cold Hanks’ Balanced Salt Solution (Sigma). The lung was excised, minced with scissors and further digested in 0.1% Collagenase B, 1% Pronase and 0.025% DNase 1 (Roche). After addition of the enzymes the tissues were incubated at 37°C for 20 min and forced through a 70 μM cell strainer. The cell suspension was centrifuged twice at 200g for 5 min. Following staining detailed in “Flow Cytometry”, pericytes were sorted using a FACSAria (BD Biosciences).

##### **Live**r

Pericyte isolation from mouse livers were carried out as described in (Graham, 2002). Briefly, livers from 4 mice were harvested in cold Hanks’ Balanced Salt Solution post perfusion. Organs were minced, enzyme digested and filtered in a similar way to the lungs. The suspension was centrifuged at 600g for 7 minutes at room temperature. A gradient was prepared using 60% Optiprep (Alere) with HBSS in the lower phase and 39.86% Optiprep in the middle phase homogenised with the cell pellet. The top was layered with 0.5 ml HBSS followed by centrifugation at 1400g for 20 minutes at 4°C without break. The interphase between the top and middle layers were collected and washed by centrifugation at 600g for 7 minutes. Finally, the pellet was treated with RBC lysis buffer (Sigma) and washed. Cells were isolated and plated in tissue culture treated 12 well plates (Corning) in pericyte medium i.e., DMEM with 20% FBS, 10mM L-Glutamine, 100units Penicillin Streptomycin (Gibco) and were allowed to settle overnight at 37°C, in humidified atmosphere at 5% CO_2_. The cells were then washed thoroughly with PBS and then treated as indicated.

#### Pericyte Culture Conditions and Treatment

Pericytes were cultured in DMEM containing glucose, pyruvate and L-glutamine (Gibco), supplemented with penicillin-streptomycin, 5 x10^-5^ M 2-mercaptoethanol (Sigma) and 15% FCS (Gibco) at 37°C in a humidified atmosphere at 5% CO_2_. Isolated pericytes were incubated in the presence of 50-100 ng/mL of AREG (BioLegend) or 0.5 ng/mL of TGF-β1 (R&D Systems). Inhibitors were used at the following concentrations: Gefitinib (LC laboratories) (1 mM), U-73122 (1 mM), CWHM12 (2 μM) and Alk5i (1 μM). CWHM-96 the inactive enantiomer of CWHM-12 was used as control. Under these conditions, cell viability was higher than 92%. After 16 hours of culture, cells were stored in TRIzol (Invitrogen) and supernatants were frozen for subsequent analysis. For the analysis of the expression of αSMA by WB, cells were cultured for 24 hours.

#### TGF**-**β Latent Associated Protein (LAP) Binding Assay

Activation of integrin-α_V_ was quantified as described before (Munger et al., 1999). In summary, pericytes in suspension were treated with 50-100 ng/mL rAREG for 0–30 min in the presence or absence of a blocking antibody against integrin-α_V_ (clone RMV-7, 1 μg/ml) and subsequently with 0.1 mg/ml recombinant mouse LAP (Biolegend) for 30 min at 37°C. After fixation and washing, cells were stained with an anti-LAP and analyzed by flow cytometry.

#### Macrophage Culture Conditions and Treatment

Bone marrow-derived macrophages (BMDMs) from WT mice were differentiated from bone marrow precursors. Briefly, bone marrow cells were isolated and propagated for 7 days in RPMI (Gibco) containing 20% FCS (Sigma-Aldrich), 30% L929 conditioned media and 1% Pen/Strep (Gibco).

Alveolar macrophages were obtained from the bronchoalveolar lavage (BAL) of mice with Dulbecco’s phosphate buffered saline with 0.5% BSA (m/v). Peritoneal macrophages were obtained from mice by washing the peritoneal cavity with RPMI 1640 containing 2 mM L-glutamine, 200U/ml penicillin, 100 μg/ml streptomycin. Cells were separated from the lavage fluid by centrifugation (250 x g, 5 min), resuspended in RPMI 1640 medium (5% heat-inactivated FBS, 100 U/ml penicillin, 100 μg/ml streptomycin, supplemented with 2 mM glutamine), and purified by adherence. Viability of adherent cells was assessed by trypan blue exclusion test. Flow cytometry analysis determined that at least 90% of adherent cell were macrophages. Subsequently, cells were treated with LPS, IFNγ, IL-4, IL-13, IL-25, IL-33, TSLP (100 ng/ml) and ATP (10 μM). After 10 hours of culture, cells were stored in TRIzol for subsequent analysis.

#### Co-cultures of Macrophages and Pericytes

Alveolar macrophages were obtained from naïve and *N. brasiliensis*-infected mice as described above. After purification, macrophages were cultured for 24 h in DMEM containing glucose, pyruvate and L-glutamine, supplemented with penicillin-streptomycin, 5 x10^-5^ M 2-mercaptoethanol and 15% FCS. After which FACS-sorted pericytes were added 1:50 and cultured for another 16 hours. Alternatively, the supernatant of the macrophages was used to culture primary pericytes.

#### Measurement of Bioactive TGF-β

##### Luciferase Assays

Bioactive TGF-β was measured in the supernatants of liver pericyte cultures using transgenic Mink Lung Epithelial cells (MLECs), which express luciferase, downstream of the (PAI-1) promoter, corresponding to the expression of bioactive TGF-β (Abe et al., 1994). The assay was carried out as described in (Wipff et al., 2007). In brief, MLECs were plated as 25,000/ well and allowed to adhere for 3 hours following which their supernatant was replaced with either TGF-β standards, or the pericyte supernatant from control or treated wells. Measurement of total TGF-β was achieved by heat activation of the samples. After 20 hours of incubation, the MLECs were lysed and were read for luciferase activity using the Varioscan Flash (Thermo Scientific). Data were represented as a percentage of the total TGF-β in the respective sample.

##### FACS-based ELISA

Alternatively, active and total TGF-β1 were measured in culture supernatants using ELISA kits (Biolegend) as per manufacturer’s instructions.

#### *Nippostrongylus brasiliensis* Infection and Delivery of Recombinant AREG and TGF-β1

Rat-adapted *N. brasiliensis* was maintained by serial passage through Sprague Dawley rats, as described previously (Sutherland et al., 2014). Mice were infected subcutaneously with 300 *N. brasiliensis* third-stage larvae and 500 larvae for SpO_2_ measurements. Analysis of samples was performed at different times post infection, as indicated. Adult worm burden was determined by removing the small intestine and exposing the lumen by dissection. The lungs were washed with Dulbecco’s phosphate buffered saline to obtain the bronchoalveolar lavage (BAL) before tissue collection. Subsequently, the right lung was fixed for histology analysis. Alternatively, one section of the left lung was stored in TRIzol for mRNA quantification; another section was homogenized to obtain single cell suspensions for flow cytometry analysis, and a third section was stored for western blot analysis. In this case, lung tissue was homogenized in lysis buffer.

In rAREG and rTGF-β1 delivery experiments, 5 μg of either mouse recombinant protein (BioLegend) were injected intraperitoneally (AREG) or via the tail vein (TGF-β1) at days 1, 2 and 3 post *N. brasiliensis* infection following procedures described previously (Jamieson et al., 2013, Monticelli et al., 2011).

#### Liver Injury

Carbon tetrachloride liver injury was induced by injecting i.p. with 0.5 μl/g body weight CCl_4_ (Sigma-Aldrich) in a 1:1 ratio in olive oil (Sigma) or olive oil alone for a control group. Livers were harvested at specific time points post injection, as indicated. For the analysis of different parameters, the liver was first perfused in situ by injecting ice cold PBS (Sigma) through the hepatic portal vein and gently cutting the inferior vena cava to release the pressure built up of blood. Different lobes of the liver were collected for cell isolation, qRT-PCR and histology. The lobes used for different readouts were consistent across all experiments.

#### Evans Blue Dye Leakage Assays

Evans Blue leakage assays were performed as described before (Goto et al., 2010): mice were injected via the tail vein with Evans Blue dye (Fluka) (100 mg/kg) four days after *N. brasiliensis* infection or three days after CCl_4_-induced liver injury. Three hours after Evans Blue injection, BAL was performed and alveolar cells were removed by centrifugation. Alternatively, the livers were perfused, removed, and immersed in formamide at 55°C. After 24 hours' incubation, formamide including eluted blue dye was collected. The absorbance at 650 nm was measured with BAL fluid and formamide samples.

#### *In Vivo* Delivery of Brefeldin A and Apyrase

Apyrase treatment was performed by intravenous administration of 25 U apyrase (Sigma-Aldrich) 24 and 32 hours prior to harvest.

In vivo delivery of brefeldin A was performed as previously described (Minutti et al., 2017a). In brief, brefeldin A (Sigma) was resuspended at 20 mg/ml in DMSO. Further dilution to 1 mg/ml was made in PBS, and 200 μl were injected intravenously 6 hours later BAL and lung tissue were harvested and processed in the presence of Monensin (Sigma) for flow cytometry analysis.

#### *In Vivo* CWHM 12 Delivery

CWHM 12 and CWHM-96, the inactive control enantiomer of CWHM-12, were prepared as reported previously (Henderson et al., 2013). CWHM 12 and CWHM 96 were solubilized in 50% DMSO (in sterile water) and dosed to 100 mg per kg body weight per day. Drug or vehicle (50% DMSO) was delivered by implantable ALZET osmotic minipumps (Charles River). Pumps were inserted subcutaneously 3 days before *N. brasiliensis* infection. Delivery of CWHM 96 or vehicle (DMSO/H_2_O) produced indiscernible effects.

#### Measurement of Pulse Oximetry

The MouseOxTM Pulse-oximeter (Starr Life Sciences) was used to measure blood SpO_2_ and breathe rate in *N. brasiliensis*-infected mice during the course of infection. A depilatory agent (Nair, Church & Dwight) was applied to the neck of mice a day prior to *N. brasiliensis* infection to remove hair and delay future hair growth. For readings mice were sedated with 2.5-5mg/kg intra-peritoneal Midazolam (Roche). Subsequently, the oximeter clip was placed on the neck and percent SpO_2_ was measured each second over several minutes, data shown is the ratio of SpO_2_/breathe rate readings recorded over 3–5 min per mouse.

#### Histology

Lung and liver lobes were fixed with 10% neutral buffered formalin (lungs were inflated with fixative agent), incubated overnight and transferred to 70% ethanol. Organs were paraffin-embedded, sectioned and stained with hematoxylin and eosin (H&E) and Periodic acid–Schiff (PAS). The following morphological changes within the lungs were graded - Alveolar wall destruction: 0 absent, 1 mild damage, 2 moderate, 3 severe; Peri-bronchial/peri-vascular mononuclear cell infiltrate and Peri-bronchial/peri-vascular neutrophil infiltrate: 0 absent, 1 <20% bronchi/vessel involvement, 2 20%–70% involvement, 3 >70% involvement; Alveolar neutrophils: 0 absent, 1 mild increase in neutrophils, 2 moderate, 3 severe). Alternatively, livers were graded according to the following criteria: Necrosis 0 no pathological change, 1 degenerated hepatocytes with only rare foci of necrosis, 2 small area of mild centrilobular necrosis around the central vein, 3 area of mild centrilobular necrosis severer than grade 2, 4 centrilobular necrosis severer than grade 3. Inflammation 0 none, 1 isolated inflammatory cells, >10% central vein profiles, 2 diffuse individual and aggregates of pericentral inflammatory cells, 3 confluent pericentral inflammation, <50% circumference, 4 confluent pericentral inflammation, >50% circumference. The degree of organ injury and inflammation was assessed by the sum of scores of the parameters, and the average sum of each was compared between groups.

#### RNA Extraction and Quantitative Real-Time PCR

Tissue or cells in culture were homogenised in TRIzol with a TissueLyser (Qiagen) and RNA was isolated following manufacturer’s instructions. Reverse transcription was performed using 1 μg of total RNA using 200 U of M-MLV reverse transcriptase, 10 mM dNTPs, and 0.5 μg Oligo dT15 and RNasin inhibitor (Promega). Transcript levels of genes of interest were measured by real-time PCR with the Lightcycler 480 II system (Roche) using Taqman Master kit and specific primers (STAR Methods; Table S1), as previously described. PCR amplification was analysed using 2nd derivative maximum algorithm (LightCycler 480 Sw 1.5, Roche) and the expression of the gene of interest was normalised to the housekeeping gene *Rn18s*.

#### Western Blots

Tissue or cell lysates were prepared by homogenization in a buffer containing: 10 mM HEPES (pH 7.9), 15 mM MgCl, 10 mM KCl, 0.5 mM EDTA, 0.2% Triton X-100, 1 mM benzamidine, 200 mg/ml aprotinin, 200 mg/ml leupeptin, 1 mM PMSF (Sigma-Aldrich) and phosphatase inhibitors: 20 mM b-glycerophosphate, 10 mM NaF, 10 mM sodium pyrophosphate, and 2 mM orthovanadate (Sigma-Aldrich). Tissue lysates were resolved by 8% (m/v) SDSPAGE in reducing conditions and transferred to nitrocellulose membranes. After blocking with 2% BSA, membranes were washed and incubated with an anti-pSMAD3, αSMA or α/β-Tubulin antibodies (Cell Signaling) overnight at 4°C. Proteins were detected with secondary Abs goat anti-mouse A680 (Life Technologies) and goat anti-rabbit IR800 (Thermo scientific) and visualized with an infrared imaging system (Odyssey; LI-COR Biosciences).

#### Flow Cytometry

Single cell suspensions from lung or liver lobes were prepared by digesting with 0.03% Collagenase D, 0.038% Collagenase B and 0.025% DNase 1 (Roche) in HBSS at 37°C for 20 minutes. Tissue was homogenized by forcing through a 70 μM cell strainer and subsequently treated with red blood cell lysis buffer (Sigma). Red blood cells and other tissue and BAL cells were counted using an automated cellometer T4 (Peqlab). Cells were incubated with Fc block (CD16/CD32 and mouse serum) (BD Biosciences) and stained with fluorescent conjugated antibodies against different cell surface antigens (STAR Methods) or isotype control, in some cases followed by secondary reagents (Invitrogen). Different cell populations were identified by the expression of markers as shown in Figure S3. Following surface staining, cells were fixed with 2% paraformaldehyde in Dulbecco’s phosphate buffered saline for 20 min at room temperature, permeabilized with Perm wash (BD Biosciences), and then stained with anti- amphiregulin, anti- RELMα, or isotype control followed by secondary reagents (Invitrogen). For detection of Ki67 and FoxP3, cells were stained for surface markers, then fixed and permeabilized using FoxP3 staining buffer set (eBioscience), and subsequently stained with FoxP3 or Ki67 set. Expression of amphiregulin, RELMα and Ki67 was determined relative to isotype control staining. Live/Dead (Life Technologies) was used to exclude dead cells from analysis. Samples were analyzed by flow cytometry using Becton-Dickinson FACS LSR II and FlowJo software.

### Quantification and Statistical Analysis

Statistical evaluation of different groups was performed either by analysis of variance (ANOVA) followed by the Tukey multiple comparison test or by non-parametric Mann-Whitney test, as indicated. An α level ≤ 5% (p ≤ 0.05) was considered significant. All statistical calculations were performed using PRISM (Graphpad).

### Data and Software Availability

All data are available in the manuscript or the Supplemental Information.

## Supplemental Information

Document S1. Figures S1–S6 and Table S1

Document S2. Article plus Supplemental Information
