# Supplementary material for: A Macrophage-Pericyte Axis Directs Tissue Restoration via Amphiregulin-Induced Transforming Growth Factor Beta Activation
Source: Immunity. 2019 Mar 19;50(3):645–654.e6. doi: 10.1016/j.immuni.2019.01.008 (PMC6436929; doi:10.1016/j.immuni.2019.01.008)
Supplement: Document S1. Figures S1–S6 and Table S1 [file mmc1.pdf]

Immunity, Volume *50*

Supplemental Information

A Macrophage-Pericyte Axis Directs Tissue Restoration via Amphiregulin-Induced Transforming Growth Factor Beta Activation

Carlos M. Minutti, Rucha V. Modak, Felicity Macdonald, Fengqi Li, Danielle J. Smyth, David A. Dorward, Natalie Blair, Connor Husovsky, Andrew Muir, Evangelos Giampazolias, Ross Dobie, Rick M. Maizels, Timothy J. Kendall, David W. Griggs, Manfred Kopf, Neil C. Henderson, and Dietmar M. Zaiss

SUPPLEMENTAL FIGURE 1

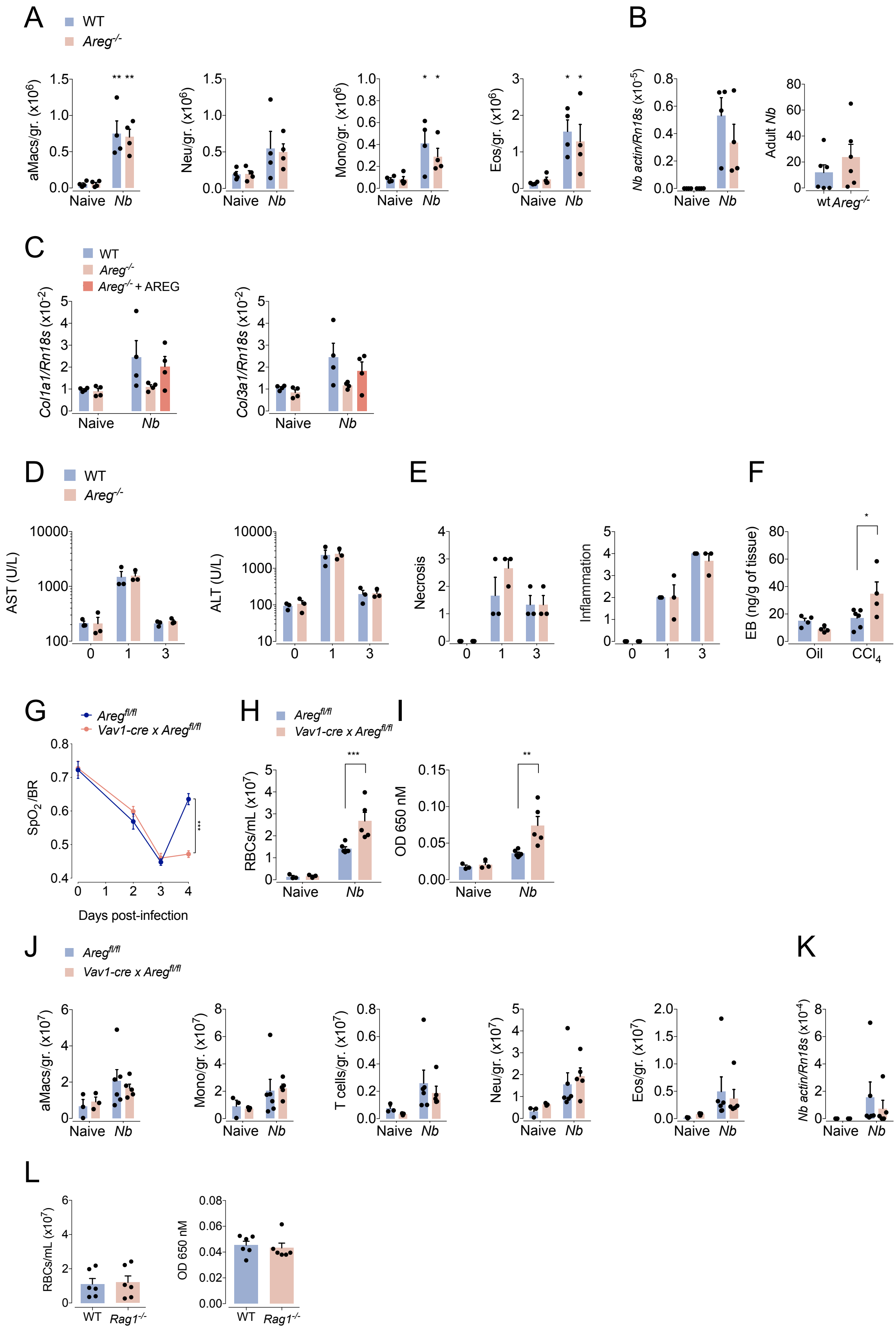

Supplemental figure 1: **Characterization of WT vs. Amphiregulin-deficient mice during acute tissue injury in lungs and liver (related to Figure 1 and 2).**

WT and *Areg*$^{-/-}$, mice were either left uninfected or infected with *Nippostrongylus brasiliensis* (a-c). In (c) 5 μg of rAREG was injected ip. at days 1, 2 and 3 post infection. Alternatively, WT and *Areg*$^{-/-}$ mice were either left untreated or liver injury was induced by intra-peritoneal injection of CCl_4 (d-f).
(a) Number of inflammatory cellular infiltrates in lung cell suspensions: alveolar macrophages, neutrophils, monocytes and eosinophils on 4 dpi.
(b) Larval load in the lungs (left graph) and adult worm counts in the small intestines (right graph) were determined at day 2 or day 6 post-infection by *Nippostrongylus*-specific actin mRNA expression or by worm count, respectively.
(c) Expression of collagen alpha 1 type I and III-encoding genes (*Col1a1* & *Col3a1*) in the lungs as determined by qRT-PCR.
(d) Quantification of alanine transaminase (ALT) and aspartate transaminase (AST) in serum at different times after challenge.
(e) Necrosis and inflammation scores assessed in H&E sections prior to and at days 1 and 3 after treatment.
(f) Extravasation of Evans blue into the liver tissue as a marker of vascular permeability on day 3 after intra-peritoneal CCl_4 injection.
All data are representative of at least two independent experiments (mean ± SEM); results for individual mice are shown as dots.

WT, *Vav1-cre x Areg*$^{fl/fl}$ and/or *Rag1*$^{-/-}$ mice were either left uninfected or infected with *Nippostrongylus brasiliensis*.
(g) Oxygen saturation in the blood at different dpi.
(h) Number of red blood cells in the BAL on 4 dpi.
(i) Extravasation of Evans blue into the alveolar space as a marker of vascular permeability on day 4 dpi.
(j) Number of inflammatory cellular infiltrates in lung cell suspension on day 4 dpi: alveolar macrophages, monocytes, T-cells, neutrophils and eosinophils.
(k) Larval load in the lungs was determined at day 2 post-infection by quantifying *Nippostrongylus*-specific actin mRNA expression.
(l) Number of red blood cells and extravasation of Evans blue into the BAL on 4 dpi.
Data represent mean ± SEM; results for individual mice are shown as dots.

SUPPLEMENTAL FIGURE 2

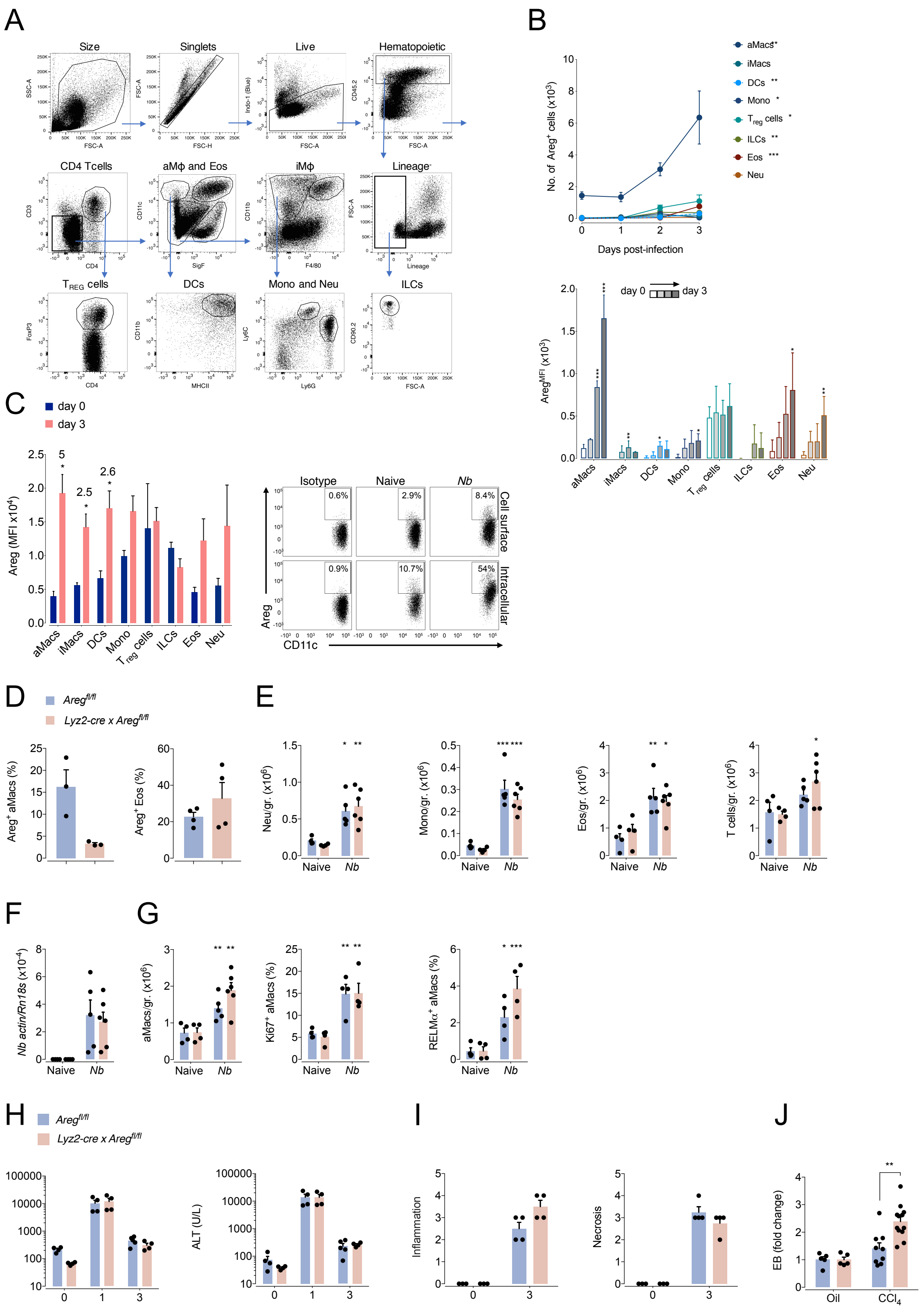

Supplemental figure 2: **Amphiregulin expression by different leukocytes following *Nippostrongylus brasiliensis* infection (related to Figure 2).**
WT mice were either left uninfected or infected with *Nippostrongylus brasiliensis*.
(a) Representative gating strategy for different leucocyte populations from lung cell suspensions.
(b) number of AREG positive and AREG MFI (cell surface staining) in different leukocytes in lung cell suspensions at different times post-infection (n = 4 mice).
(c) Amphiregulin MFI (cell surface and intracellular) in different leukocyte populations following i.v. injection of Brefeldin-A at 3 dpi. A representative dot plot is shown comparing cell surface vs. total (following 6 hrs *in vivo* Brefeldin-A treatment) AREG staining.
All data are representative of at least two independent experiments (mean ± SEM).

WT and *Lyz2-cre x Areg*$^{fl/fl}$, mice were either left uninfected or infected with *Nippostrongylus brasiliensis* (d-g). Alternatively, WT and and *Lyz2-cre x Areg*$^{fl/fl}$ mice were either left untreated or liver injury was induced by intra-peritoneal injection of CCl_4 (h-j).
(d) Cell surface Amphiregulin expression by alveolar macrophages and eosinophils showing targeted deletion of Amphiregulin in alveolar macrophages.
(e) Number of inflammatory cellular infiltrates in lung homogenates on 4 dpi: neutrophils, monocytes, eosinophils and T-cells.
(f) Larval load in the lungs was determined at day 2 post-infection by quantifying *Nippostrongylus*-specific actin mRNA expression.
(g) Absolute number of alveolar macrophages in lung homogenates and the expression of markers of proliferation (Ki67) and alternative activation (RELMα) before and 4 dpi with *Nippostrongylus brasiliensis*.
(h) Quantification of alanine transaminase (ALT) and aspartate transaminase (AST) in serum at different times after challenge.
(i) Necrosis and inflammation scores assessed in H&E sections prior to and at days 1 and 3 after treatment.
(j) Extravasation of Evans blue into the liver tissue as a marker of vascular permeability on day 3 after intra-peritoneal CCl_4 injection.
All data are representative of at least two independent experiments (mean ± SEM); results for individual mice are shown as dots.

SUPPLEMENTAL FIGURE 3

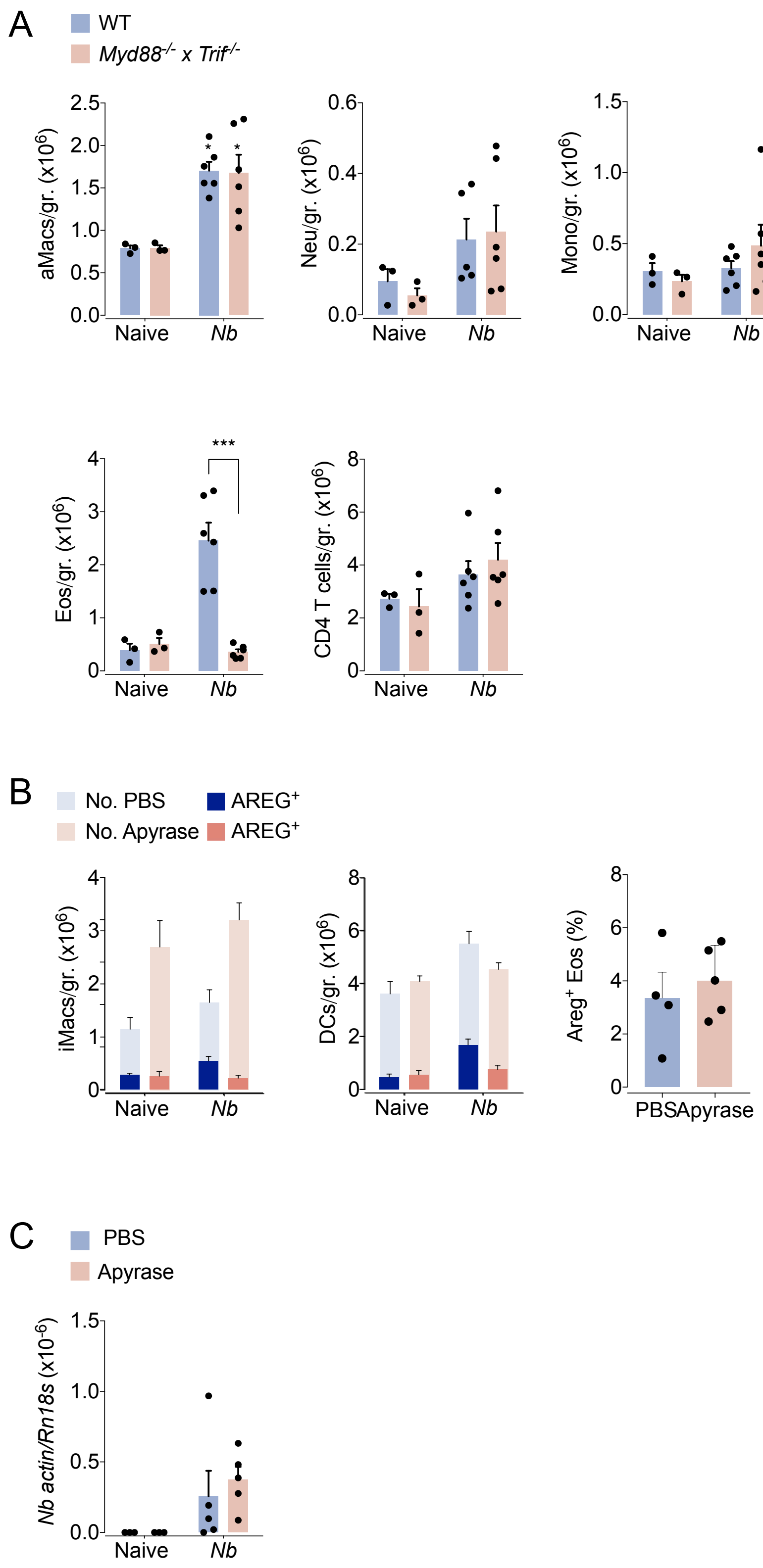

Supplemental figure 3. ATP but not LPS or IL-33 drives Amphiregulin expression by mononuclear phagocytes (related to Figure 3).

WT and *Myd88-/- x Trif-/-* mice were either left uninfected or infected with *N. brasiliensis*. For some experiments WT mice received two doses of 25 U of Apyrase (second dose after 8 hours) at day 1 post-infection.

(a) Number of inflammatory cellular infiltrates in lung homogenates on 4 dpi: alveolar macrophages, neutrophils, monocytes, eosinophils and T-cells.

(b) Number and relative expression of Amphiregulin (cell surface and intracellular) by interstitial macrophages and dendritic cells following i.v. injection of Brefeldin-A at 2 dpi (left). Cell surface expression of Amphiregulin by eosinophils was also analysed at 2 dpi (right).

(c) Larval load in the lungs was determined at day 2 post-infection by quantifying *Nippostrongylus*-specific actin mRNA expression.

All data are representative of at least two independent experiments (mean ± SEM); results for individual mice are shown as dots.

SUPPLEMENTAL FIGURE 4

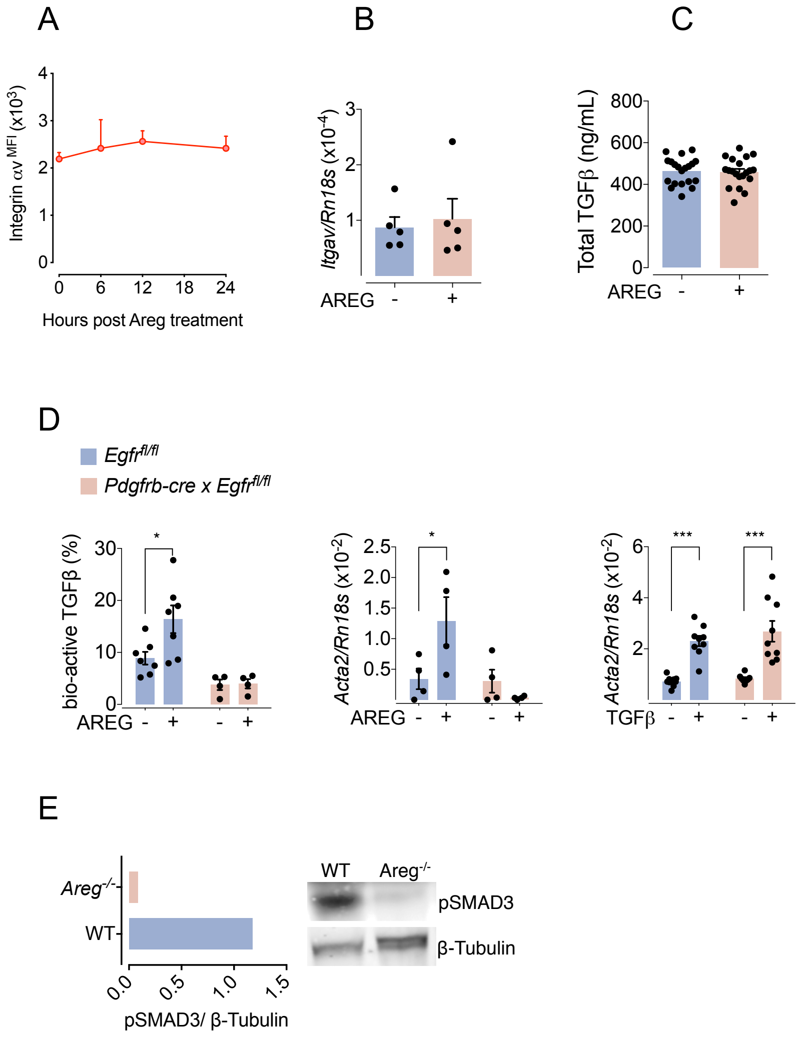

Supplemental figure 4: Amphiregulin induces the integrin-α_v mediated activation of TGF-β by pericytes (related to Figure 4 and 5).

Liver (a, d) or lung (b-c) pericytes, isolated from WT, *Pdgfrb-cre x EGFR*$^{fl/fl}$ and *Pdgfrb-BAC-eGFP* mice, were cultured in the presence of 100 ng/ml rAREG or 0.5 ng/mL TGF-β.

(a) Expression of integrin-α_v on the cell surface of cultured cells was determined by FACS at different times after treatment. Data are representative of four individual preparations in parallel.

(b) Transcriptional levels of integrin-α_v-encoding gene as measured in pericyte cultures 24 hours after treatment by qRT-PCR.

(c) Total amount of TGF-β was measured by ELISA in the supernatants of pericyte cultures.

(d) Release of bio-active TGF-β from primary liver pericytes derived from WT and *Pdgfrb-cre x EGFR*$^{fl/fl}$ mice after 24 hrs of Amphiregulin treatment was measured using MLEC or the differentiation of treated pericytes was measured by the mRNA expression of αSMA using qRT-PCR.

All data are representative of at least two independent experiments (mean ± SEM); results for individual pericyte preparations are shown as individual dots.

(e) WT and *Areg*$^{-/-}$ mice were infected with *Nippostrongylus brasiliensis* and phosphorylation of SMAD3 was assessed in lung homogenates by Western blot at 4 dpi. Values are normalized by total β-Tubulin.

SUPPLEMENTAL FIGURE 5

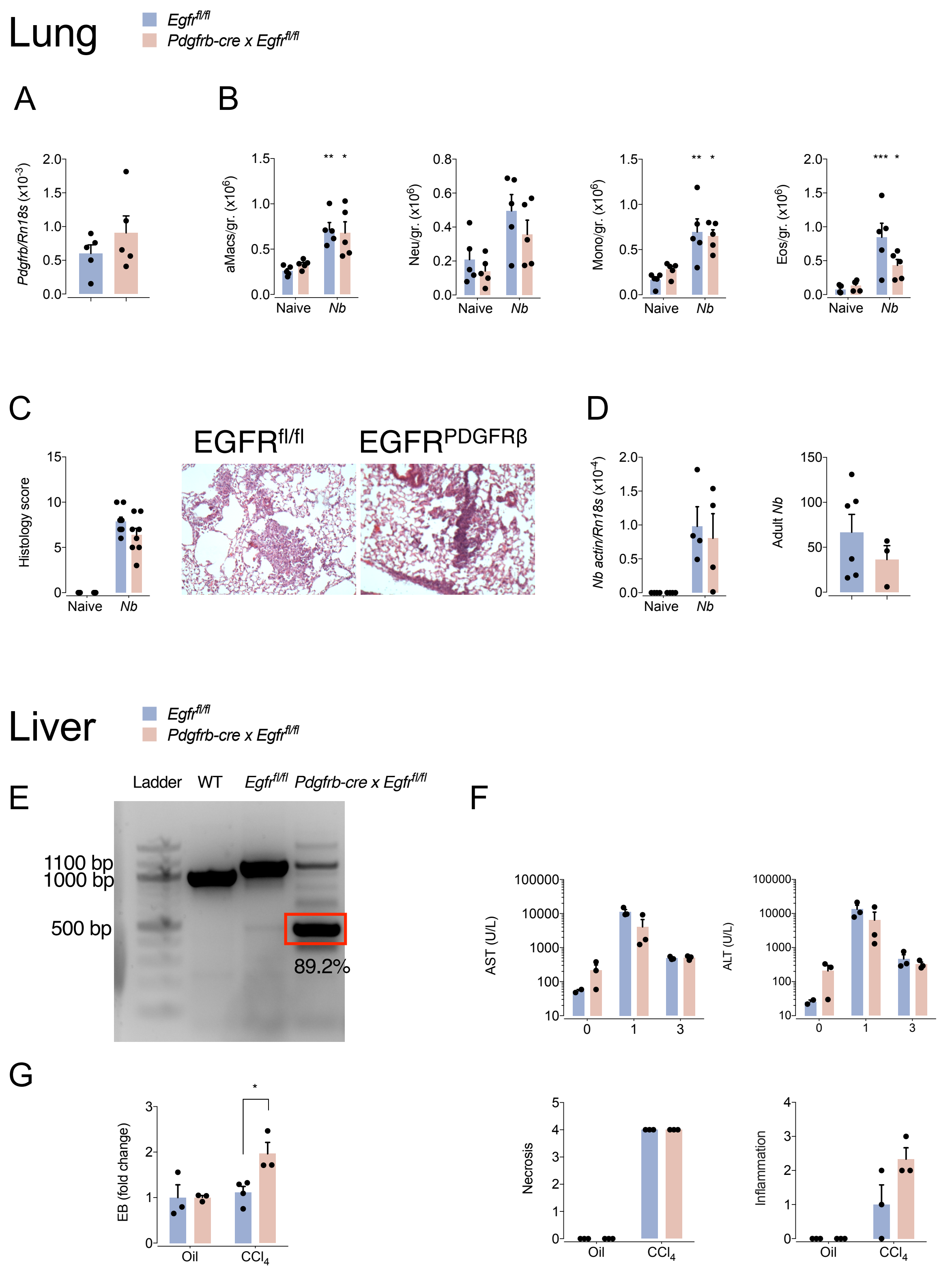

Supplemental figure 5: Characterization of WT vs. pericyte-specific EGFR-deficient mice during acute tissue injury in lungs and liver (related to Figure 5).

Egfr$^{fl/fl}$ or *Pdgfrb-cre x EGFR*$^{fl/fl}$ mice were either left uninfected or infected with *Nippostrongylus brasiliensis*.

(a) Expression of PDGFRβ-encoding gene as an indication of the stability of the pericyte population was determined in *Egfr*$^{fl/fl}$ or *Pdgfrb-cre x EGFR*$^{fl/fl}$ mice at steady state.

(b) Number of inflammatory cellular infiltrates in lung cell suspensions: alveolar macrophages, neutrophils, monocytes and eosinophils.

(c) Representative H&E staining of lung tissue (x100) and ALI scores at days 0 and 4 after inoculation.

(d) Larval load in the lungs (left graph) and adult worm counts in the small intestines (right graph) were determined at day 2 or day 6 post-infection by *Nippostrongylus*-specific actin mRNA expression or by worm count, respectively.

(e) Liver pericytes were isolated from C57BL/6, *Egfr*$^{fl/fl}$ or *Pdgfrb-cre x EGFR*$^{fl/fl}$ mice and differentiated into myo-fibroblast *in vitro* subsequently, the cre translocase-induced alterations in the EGFR gene locus were detected by PCR.

Egfr$^{fl/fl}$ or *Pdgfrb-cre x EGFR*$^{fl/fl}$ mice were either left untreated or liver injury was induced by intra-peritoneal injection of CCl_4.

(f) Quantification of alanine transaminase (ALT) and aspartate transaminase (AST) in serum at different times after challenge (top) and necrosis and inflammation scores assessed in H&E sections prior to and at days 1 and 3 after treatment (bottom).

(g) Extravasation of Evans blue into the liver tissue as a marker of vascular permeability.

All data are representative of at least two independent experiments (mean ± SEM); results for individual mice are shown as dots.

SUPPLEMENTAL FIGURE 6

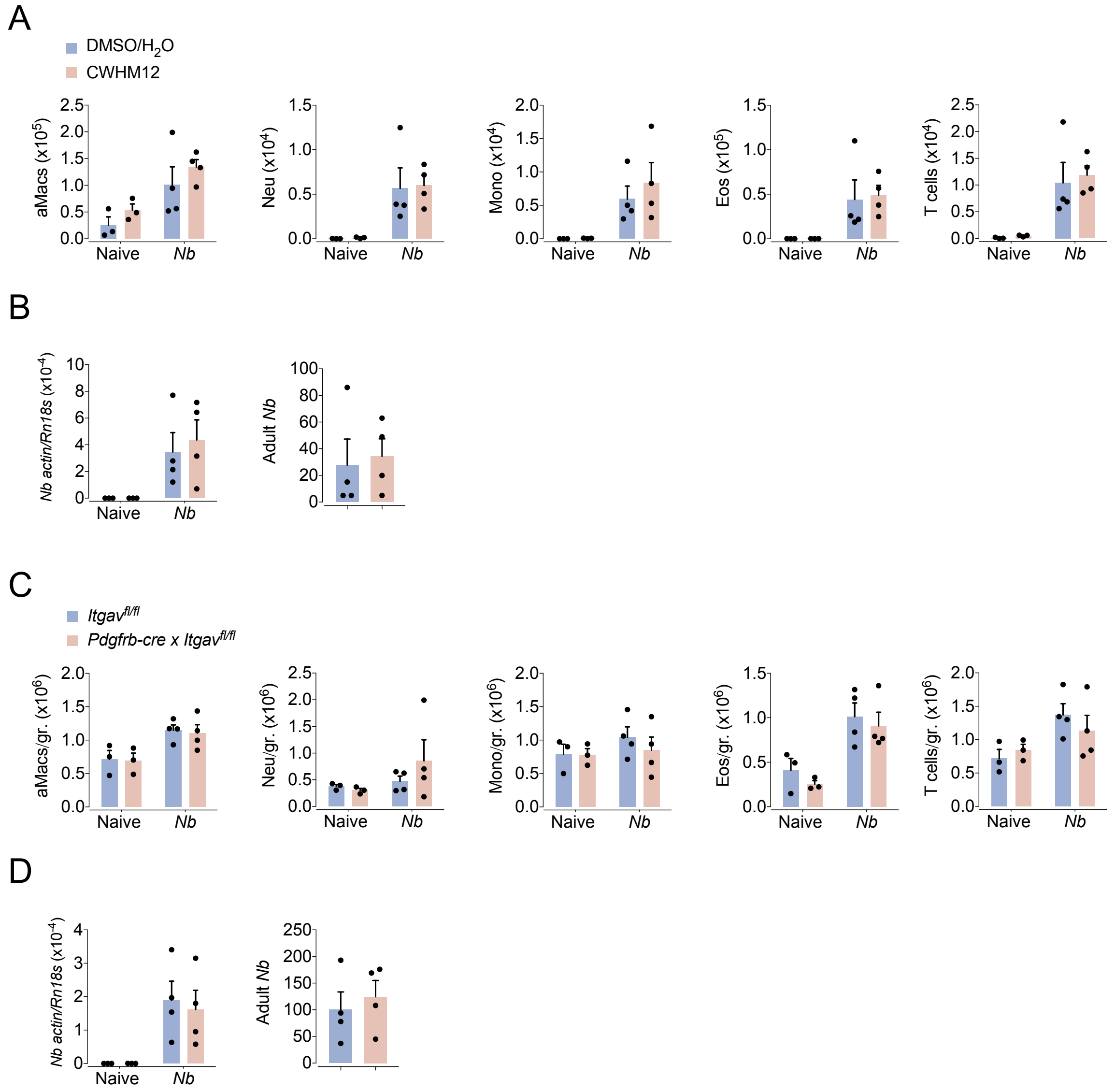

Supplemental figure 6: Characterization of pharmacological inhibition or pericyte-specific ablation of integrin αv during acute lung injury caused by *Nippostrongylus brasiliensis* infection (related to Figure 6).

WT and/or *Pdgfrb-cre x Igtav*$^{fl/fl}$ mice were either left uninfected or infected with *Nippostrongylus brasiliensis.* In (a-b) mini-pumps containing the integrin-αv inhibitor CWHM12 were inserted subcutaneously in WT mice 3 days prior to infection.

(a, c) Number of inflammatory cellular infiltrates in the BAL (a) or lung cell suspensions (c): alveolar macrophages, neutrophils, monocytes, eosinophils and T-cells was determined 4 dpi.

(b, d) Larval load in the lungs (left graph) and adult worm counts in the small intestines (right graph) were determined at day 2 or day 6 post-infection by *Nippostrongylus*-specific actin mRNA expression or by worm count, respectively.

All data are representative of at least two independent experiments, except for d&e (mean ± SEM); results for individual mice are shown as dots.

Supplemental table 1: PCR Primers, Related to STAR Methods

Gene	Primer	Sequence
Aregflox/flox	Areg 5-arm	CCAGCTTTCTCCACCTCAAG
	Areg Crit	TGGACCTCGCATGACATAGA
	5 mut-R1	GAACTTCGGAATAGGAACTTCG
LysM-Cre	66 (cre)	CCC AGA AAT GCC AGA TTA CG
	67 (common)	CTT GGG CTG CCA GAA TTT CTC
	68 (WT)	TTA CAG TCG GCC AGG CTG AC
Endogenous CD11c	For	GACAACTTCCCTCCTGGTCTCTG
	Rev	CATCCAAGTTGAAGCCAAGACAA
CD11c-Cre transgene	For	GACAACTTCCCTCCTGGTCTCTG
	Rev	CCCAGAAATGCCAGATTACG
Egfrflox/flox	EGFR Fwd1	AAG TTT AAG AAA CCC CGC TCT ACT
	EGFR R4 Fwd	GCC TGT GTC CGG GTC TCG TCG
	EGFR R6 Rev	CAA CCA GTG CAC CTA GCC TGG C
Egfr del	FRT4	CTA TGC CTA AGA GGC GGA ATA
	Con1	CAT TCC ACA GCT TCA AGT ACT CAT TC
Cre (for Pdgfrb-Cre)	For	TGC CAC GAC CAA GTG ACA GCA
	Rev	AGA GAC GGA AAT CCA TCG CTC
Vav1-Cre	For	AGA TGC CAG GAC ATC AGG AAC CTG
	Rev	ATC AGC CAC ACC AGA CAC AGA GAT C
Itgavflox/flox	Intav4f6	GGT GAC TCA ATG TGT GAC CTT CAG C
	Intav5b3	CAC AAA TCA AGG ATG ACC AAA CTG AG
Nb Actin	For	ACGACGTGGCAGCTCTCGTTGTGG
	Rev	GGTGCTTCGGTCAGCAGCACGGGA
